# Stress susceptibility in *Trypanosoma brucei* lacking the RNA-binding protein ZC3H30

**DOI:** 10.1371/journal.pntd.0006835

**Published:** 2018-10-01

**Authors:** Chaitali Chakraborty, Christine Clayton

**Affiliations:** Zentrum für Molekular Biologie, Universität Heidelberg, Heidelberg, Germany; Liverpool School of Tropical Medicine, UNITED KINGDOM

## Abstract

Trypanosomes rely on post-transcriptional mechanisms and mRNA-binding proteins for control of gene expression. *Trypanosoma brucei* ZC3H30 is an mRNA-binding protein that is expressed in both the bloodstream form (which grows in mammals) and the procyclic form (which grows in the tsetse fly midgut). Attachment of ZC3H30 to an mRNA causes degradation of that mRNA. Cells lacking ZC3H30 showed no growth defect under normal culture conditions; but they were more susceptible than wild-type cells to heat shock, starvation, and treatment with DTT, arsenite or ethanol. Transcriptomes of procyclic-form trypanosomes lacking ZC3H30 were indistinguishable from those of cells in which ZC3H30 had been re-expressed, but un-stressed bloodstream forms lacking ZC3H30 had about 2-fold more *HSP70* mRNA. Results from pull-downs suggested that ZC3H30 mRNA binding may not be very specific. ZC3H30 was found in stress-induced granules and co-purified with another stress granule protein, Tb927.8.3820; but RNAi targeting Tb927.8.3820 did not affect either ZC3H30 granule association or stress resistance. The conservation of the *ZC3H30* gene in both monogenetic and digenetic kinetoplastids, combined with the increased stress susceptibility of cells lacking it, suggests that ZC3H30 confers a selective advantage in the wild, where the parasites are subject to temperature fluctuations and immune attack in both the insect and mammalian hosts.

## Author summary

RNA-binding proteins are essential for control of gene expression in trypanosomes and leishmanias. ZC3H30 is a protein which was shown to bind to mRNAs; when it is attached to an mRNA, that mRNA is degraded. Procyclic-form trypanosomes that lack ZC3H30 grow normally under standard conditions, but were susceptible to various stresses: heat shock, and treatment with ethanol, a reducing agent, or arsenite. ZC3H30 interacts with one other protein, and both proteins migrate to RNA-protein granules after heat shock or starvation. These results, combined with others, suggest that ZC3H30 is involved in mRNA protection after stress. The conservation of the *ZC3H30* gene in related parasites, including those that infect only arthropods, combined with the increased stress susceptibility of cells lacking it, suggests that ZC3H30 confers a selective advantage in the wild, where the parasites are subject to temperature fluctuations and immune attack in both the insect and mammalian hosts.

## Introduction

The African trypanosomes *Trypanosoma brucei*, *T*. *congolense* and *T*. *vivax* infect mammals and Tsetse flies. Their major impact is on domestic livestock, for which it was recently estimated that trypanosomiasis elimination could bring a benefit of 2.5 billion US dollars [1]. The human disease, caused by variants of *T*. *brucei*, is currently under control, with only a few thousand known cases per year [2], but resurgence from both animal [3] and human [4] reservoirs is a constant danger. In mammals, the "bloodstream form" parasites (abbreviated to BS in some Figures) live free in the blood, generating ATP from glucose by substrate-level phosphorylation and evading the immune response through changing of the major surface protein, Variant Surface Glycoprotein (VSG). In the Tsetse fly midgut, "procyclic form" trypanosomes (abbreviated to PC in some Figures) have a surface coat of procyclins (with EP or GPEET repeats) and generate energy from amino acids via mitochondrial pathways [5].

Trypanosomes and other Kinetoplastids are unusual in that they lack control of transcription initiation at the level of individual protein-coding genes. RNA polymerase II transcription is polycistronic, initiating at GT-rich regions that are marked by specific chromatin modifications [6–8]. Transcription units can contain more than 100 genes, which are mostly unrelated in both function and regulation. Individual mRNAs are excised by *trans* splicing of a capped 39 nt spliced leader (*SL*), and by polyadenylation [9, 10]. This peculiar genome arrangement means that regulation of protein-coding gene expression depends almost exclusively on control of mRNA processing, translation, and decay [11, 12]. RNA-binding proteins play major roles in this regulation [13–18].

Within their hosts, trypanosomes are subjected to a variety of stresses: temperature variations due to fever in the mammal and changes in ambient temperature in tsetse; and assaults from the adaptive and innate immune systems. Like all other organisms, trypanosomes respond to stresses by shutting down expression of all genes except those required for immediate survival. Transcription is arrested, and translation is suppressed [19]. During starvation and heat shock, most mRNAs are initially sequestered in stress granules [19–22] which—as in yeast and mammalian cells [23]—contain translation initiation factors, RNA binding proteins, RNA helicases including DHH1, some RNA degradation enzymes, and the aggregation-promoting protein SCD6 [24]. It is thought that eukaryotic stress granules may act to temporarily protect mRNAs during stress, enabling their reactivation when normal conditions are restored [23]. During heat shock in *T*. *brucei*, synthesis of some proteins, including the subset of chaperones that is required for protein re-folding, continues [19], with preferential exclusion of the relevant mRNAs from granules [25]. The heat-shock response requires the RNA-binding protein ZC3H11, which binds to and stabilises chaperone mRNAs [26]. Correspondingly, expression of ZC3H11 increases upon heat shock, through a combination of new translation and protein stabilisation [27]. Genes encoding chaperones are also preferentially located towards the end of transcription units, which means that they are the last to be affected when transcription initiation is suppressed [28]. The trypanosome response to endoplasmic reticulum stress is also unique. Inhibition of protein import, via RNAi or treatment with DTT, initiates a regulatory cascade [29] which leads to a shut-down of *SLRNA* transcription [30, 31], complete cessation of mRNA processing, and finally to death of the parasites.

RNA-binding proteins can actively cause mRNA decay, for example by interacting with components of the RNA degradation machinery (e.g. [32]), or can enhance expression, for example by recruitment of translation initiation factors or poly(A) binding protein [33]. Other RNA-binding proteins act by preventing the binding of decay-promoting or stabilizing proteins [34]. "Tethering" is a useful technique to investigate the *active* effects of proteins on mRNA fate [35, 36]. The experimental system has two components, each of which is expressed in the cell type of interest. One is the protein under investigation, which is expressed as a fusion with a highly specific RNA-binding domain such as the lambdaN peptide [37]. The other is a reporter mRNA, which has several RNA-binding domain target sites in its 3'-UTR. For the lambda N peptide, this is the "boxB" sequence. We used tethering to screen for proteins that can actively affect mRNA fate in bloodstream-form trypanosomes [38]. In addition, all proteins that could be UV-cross-linked to bloodstream-form mRNAs were identified [39]. This gave us a useful list of proteins that can both bind to mRNA, and influence mRNA fate.

ZC3H30 (Tb927.10.1540) was identified as an mRNA-binding protein that suppressed reporter expression in the tethering screen [38]. ZC3H30 was found in purified procyclic-form starvation granules [24], and by microscopy, an N-terminally GFP-tagged version [40] was predominantly cytosolic and located in granules in stressed procyclic forms (http://tryptag.org) [41]. The *ZC3H30* mRNA is present at roughly similar levels (about 1 per cell) in bloodstream and procyclic forms, and the mRNAs in both forms show similar ribosome occupancies [42–44]. In one mass spectrometric analysis of total cell lysates, ZC3H30 was not seen [45, 46]; in a second, low levels were detected in lysates of procyclic, but not bloodstream-form, parasites [45, 46]. Results of an RNAi screen suggested that in bloodstream forms, loss of ZC3H30 leads to defective cell multiplication [47]. We therefore decided to investigate its function in more detail.

## Methods

### Trypanosome cell culturing and plasmids

All experiments were done using Lister strain 427 monomorphic bloodstream form parasites expressing the Tet-repressor, and with procyclic forms expressing the tet repressor and T7 polymerase [48]. Procyclic forms were cultured in MEM-Pros Medium at 27°C and bloodstream form parasites were cultured at 37°C in HMI-9 medium [48]. Details of plasmids are in S1 Table, which also includes the selective drug concentrations. Source plasmids for tandem affinity purification, V5 tagging, YFP tagging and tethering were described in [49–52]. Once the complete deletion of the ZC3H30 genes was confirmed for the DKO cells, they were grown without blasticidin and puromycin. Prior to phenotypic assays, all cells were grown without any selective drugs for three days, but tetracycline was included in the DKO+ cultures for this period.

To measure susceptibility to hygromycin, 200mL of procyclic-form trypanosomes were inoculated on a 96-well plate at a density of 2x10^5^ cells/mL in medium without any selective drugs, and serially diluted hygromycin was added. The cells were allowed to proliferate and grow for 72 hours, then Resazurin (44 μL) (Sigma-Aldrich) was added. After 4 hours of incubation fluorescence (544 nm excitation and 590 nm emission) was measured a using FLUOstar Omerga plate reader (MBG Labtech). Growth inhibition was analysed using Variable Slope model. For bloodstream forms, the method was similar but the cells were assayed with G418 (because all lines lacked resistance) and the starting density was 4000/mL.

Trypanosomes were subjected to heat shock (41°C or 42°C for bloodstream forms, 39°C or 41°C for procyclic forms), oxidative stress by sodium arsenite (10 or 20 μM), and ethanol stress (1% or 2%) for 1 hour, followed by centrifugation (850 x g, 8 minutes). The supernatant was discarded and the cells were resuspended in media without drugs for recovery at the relevant normal growth temperature for 48 hours. For the 37°C heat shock experiment, the schedule was slightly different: procyclic cultures were diluted in pre-warmed MEM medium (37°C) at a density of 6x10^5^ cells/mL. This culture was shifted to 37°C for 1 hour, followed by addition of pre-warmed MEM-Pros medium (27°C) such that the final density of the culture was 3x10^5^ cells/mL; it was then allowed to recover at 27°C for 48 hours.

For starvation, log-phase procyclic-form trypanosomes were harvested (850xg, 8 minutes); the supernatant was discarded and the cell pellet was resuspended in 1xPBS for 5 hours at a density of 10^6^ cells/mL. Following the incubation the cultures were centrifuged as above and the cell pellet was resuspended in 10mL of MEM-Pros medium (final density - 3x10^5^ cells/mL) and allowed to recover at normal growth temperature for 48 hours. Trypanosomes were subjected to translational stress with hygromycin and G418 as shown on the relevant Figures.

### Antibodies

Antibodies were as follows; dilutions mentioned are for Western blotting. They were 〈XRND [53]; V5 (AbD sero Tec, 1:1000), GFP-tag (Roche, 1:1000), myc-tag (Santa Cruz, 1:1000), aldolase (rabbit, 1:50,000) [54], SCD6 (rabbit, 1:5000) [19], Dhh1 (rabbit, 1:10000) [19], trypanothione reductase (rabbit, 1:1000) a kind gift from Prof. Luise Krauth-Siegel; prepared by EuragenTech, Luise Krauth-Siegel personal communication), ZC3H11 (rat, 1:1000) [27], peroxiredoxin (TxNPx, rabbit, 1:1000) [55], ribosomal protein S9 (rat, 1:1000); Protein A (Sigma, 1:50,000), PAP (Sigma, 1:5000) and CAT (Sigma, 1:5000).

### Cell fractionation and protein analyses

About 2x10^8^ bloodstream form cells expressing N-terminally TAP-tagged ZC3H30 were harvested (850 xg, 10minutes) washed once in 1mL of trypanosome homogenisation buffer (THB: 25 mM Tris-HCl pH 7.8, 1 mM EDTA, 10% Sucrose, 5 μg/mL Leupeptin). Total suspension from 5x10^6^ cells was reserved. The remaining cells were lysed using silicon carbide [56] and an organellar pellet made by centrifugation (16000 xg, 15 minutes). The pellet was resuspended in 0.5 mL of THB buffer, detergent (final concentration 0.1% Igepal) was added, and the mixture was passed through a 21G syringe 20 times. After a repeat centrifugation, pellets (insoluble fraction) and supernatant (soluble organellar and nuclear proteins) were retained. For Western blotting, 5x10^6^ cell-equivalents from cytosolic (C), organellar supernatant and pellet fractions were boiled in loading buffer and subjected to SDS-PAGE.

Granules from healthy proliferating or heat shocked (41°C) trypanosomes were prepared as previously published [24, 25]. Co-immunoprecipitation assays were performed as previously described [57], using 10^8^ cells as starting material.

Labelling with [^35^S]-L-methionine was approximately as described in [54]. 5x10^6^ cells were harvested by centrifugation (850 xg, 8 min) and washed twice with 1 mL of 1x PBS at room temperature. The cell pellet was resuspended in 0.5 mL labelling medium (modified DMEM from Gibco, lacking L-methionine), and incubated for 30 min. subsequently, [^35^S]-L-methionine (10⎧Ci) was added and cells were incubated for a further 30 min. For heat shock experiments, cells were pre-incubated for 30 min. at elevated temperatures (41°C for bloodstream forms, 39°C for procyclic forms), then [^35^S]-L-methionine was added and incubation continued at the same temperatures. After incorporation of radioactive L-Methionine, the cells were centrifuged (850 xg, 5 min.); washed twice with 1mL of 1x PBS, and resuspended finally in 15 μL of Laemmli buffer. The samples were then loaded on a 10% SDS-PAGE gel. After separation of the proteins, the gel was fixed (gel fixation solution-50% Methanol, 10% glacial acetic acid, 40% distilled water, 45 min) stained with soluble Coomassie (Coomassie R250, 2 hours) followed by overnight de-staining (de-staining solution- 5% Methanol, 7.5% acetic acid). After destaining, the gel was vacuum dried, for 1 hour at 80°C. Protein synthesis under specific conditions (heat shock or translation inhibition) was analysed by autoradiography.

### RNA methods

Total RNA was extracted using peqGOLDTrifast (Peqlab). Northern blots were probed with [^32^P]-labelled DNA from *CAT*, *HSP70* (Tb927.11.11330), α-tubulin (Tb927.1.2370) and *7SL RNA* genes. MultiGauge and Adobe Photoshop were used for quantification. For RNA-Seq, Total RNA from 5 x 10^7^ procyclic DKO and DKO+ cells was isolated. 5 μg of total RNA was subjected to rRNA depletion. The integrity of mRNAs was then checked by Northern blotting using a probe that detects the α-tubulin mRNA. All sequences are available at Array Express with accession number E-MTAB-6281. Raw mapped read counts [58] are also in the supporting material. Data and datasets used and/or analysed during the current study are also available from the corresponding author on reasonable request. Subsequent analyses were done using a list of unique open reading frames (modified from [59]). Differences in mRNA abundance were assessed using DESeqU1 [60], a custom version of DESeq2 [61].

### Tandem affinity purification, mass-spectrometry and RNA

For identification of interacting proteins and RNAs, about 10^10^ procyclic form trypanosomes were harvested. To detect protein-protein interactions, we used tandem affinity purification exactly as described previously [49, 62]. For three purifications, 200μg/mL RNase A was included in the lysis and wash buffers [26] and for three further purifications, no RNAse was included. Eluates were run 1cm into a 10% SDS-PAGE resolving gel and stained with Coomassie. The protein-containing gel area was then sliced in 5 pieces, followed by tandem mass spectrometry. Samples were measured on QE Orbitrap HF (Thermo Scientific) coupled to a nanoLC system (DIONEX Ultimate 3000, Thermo Scientific) using self-packed coloumns (ID 75 mikrom, length: 25 cm) filled with Reprosil-Pur 120 C18-AQ, 1,9 mikrom (Maisch). 5ul sample was Injected on column and peptides were eluted with a gradient starting from 97% Eluent A (98.9%water, 1%ACN, 0.1%FA) and 3% B (10%water, 89.9%ACN, 0.1%FA) changing linear to 30% B in 100 minutes. Data were acquired in a top 15 method. The database search was done with mascot 2.2.0.81 using 2 databases: TriTryDB-81 TREU927 (containing 9976 sequences) and contaminants (containing 244 sequences). Trypsin was selected as protease. 2 missed cleavages were allowed. Static modification of C (Carbamidomethyl) and variable modification of N (Deamidation), M (Oxidation) and N-term (Acetyl) were User. Parent mass tolerance was set to 10 ppm and fragment mass tolerance to 20 ppm.

To identify interacting mRNAs, 10^9^ procyclic cells were harvested, resuspended in MEM-Pros medium without FCS, spread on a 27 cm diameter Petri dish and UV-cross-linked (400mJ/cm^2^) in a Stratalinker. The cells were resuspended in 1 mL of tandem affinity purification lysis buffer (TAP-lysis buffer: 10 mM Tris-HCl pH7.8, 10 mM NaCl, 0.1% Triton X-100, 5μg/mL Leupeptin) including RNase inhibitors (283⎧M Vanadyl ribonuceloside complexes, 40 U/μl RNasin). The cells were lysed by passing through a 21G syringe for 50 times, followed by centrifugation at 16,000 x g for 10 minutes. The salt concentration was adjusted to 150mM NaCl then the lysate was incubated with IgG conjugated Sepharose beads and incubated for 2 hours at 4°C. The unbound fraction was retained, the beads were washed (5x) and the eluate was recovered by protease cleavage by Tobacco etch virus (TEV) protease in TEV cleavage buffer (TAP wash buffer adjusted to 0.5mM EDTA and 1mM DTT) for 1 hour at room temperature. RNA bound to cross-linked protein was recovered by 20 μg Proteinase K treatment at 42°C for 15 minutes. RNA isolation was then done using Trifast (Peqlab). Samples from total lysate, unbound fraction and eluate were collected from each step for protein and RNA analysis. The ribosomal RNA was depleted from the unbound fraction by hybridisation with oligonucleotides and RNase H digestion, and samples were subjected to high throughput sequencing. Data are available under the accession number E-MTAB-6281. To identify bound mRNAs, standard programmes such as DeSeq cannot be used. This is because they assume that most mRNAs are similar in both compared datasets, which is not necessarily true for purifications. We therefore first calculated reads per million (RPM) for all analysed datasets. The results for one unbound or flow-through fraction, FL4, were similar to those from DKO+ total RNA and were retained for further analysis (S3 Table). The results for the other unbound fraction (FL1) were however discarded because they correlated extremely poorly with both the other unbound fraction (FL4) and with total DKO+ RNA (S3 Table). mRNAs were classified as bound if, for both eluate replicates, the RPM values were at least twice those from the FL4 and both DKO+ total preparations.

## Results

### ZC3H30 suppresses expression of a tethered mRNA

**Fig 1:**
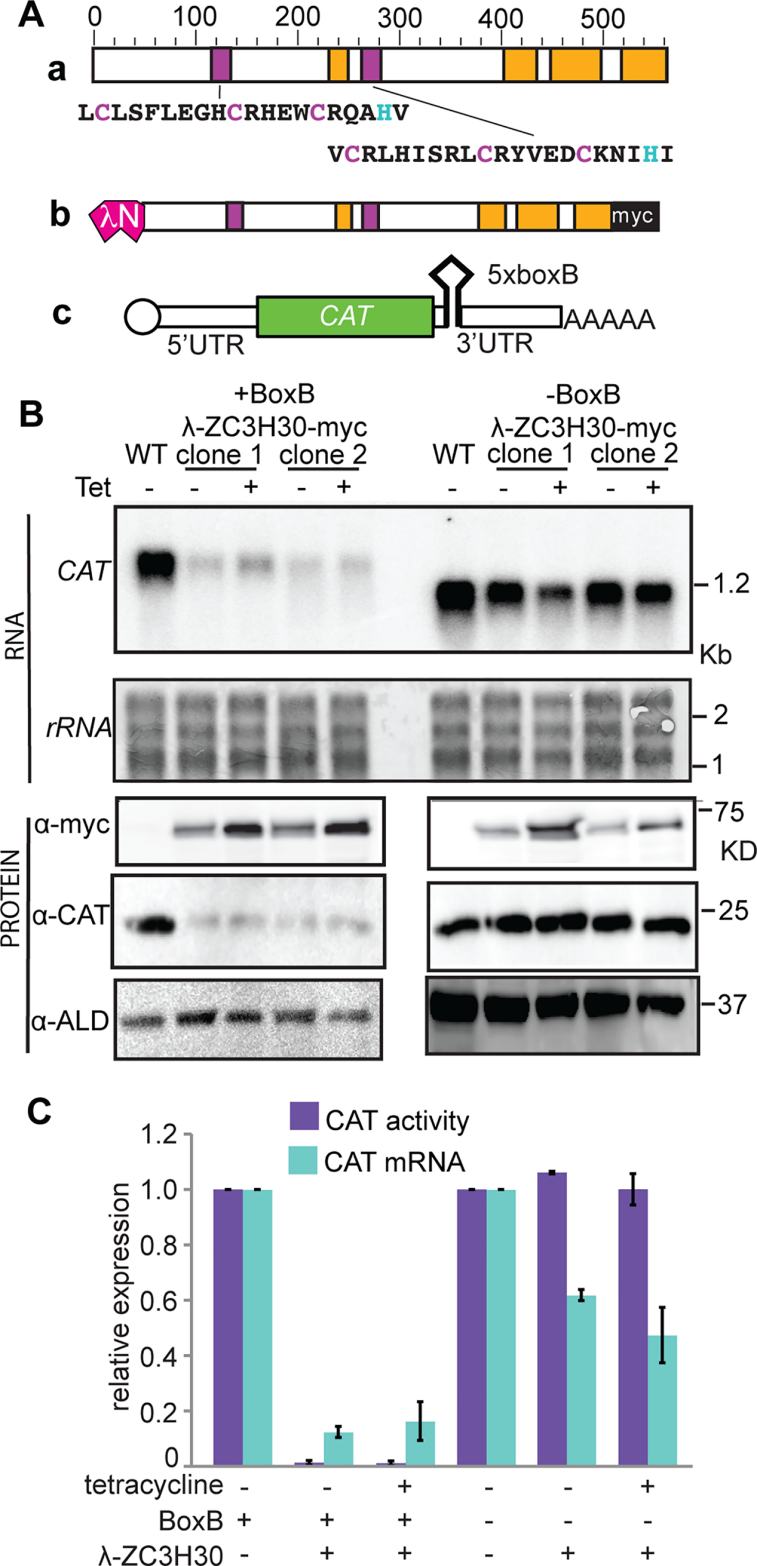
Tethering of ZC3H30 to a reporter mRNA represses expression. (A) Protein and reporter. a) Diagram of ZC3H30 protein, to scale, with zinc fingers in dark magenta and predicted disordered regions in orange. The zinc fingers are shown with the Cys and His residues in colour. b) lambdaN-ZC3H30-myc protein; c) The *CAT-boxB* mRNA. (B). RNA and protein from cells expressing *CAT* reporters with or without BoxB and λN-ZC3H30-myc. Tetracycline (100ng/mL) was added for 24h prior to analysis. For *CAT* mRNA quantification the rRNA bands (stained with methylene blue) were used as the loading control. (C) Quantification of CAT activity and *CAT* mRNA for 2 independent experiments, each with two different clones each with or without boxB (4 data points per condition). Results are expressed relative to cells with no lambdaN-ZC3H30-myc, as arithmentic mean and standard deviation for the four measurements.

ZC3H30 is a 60 kDa, 564-residue protein. In its N-terminal half it has two sequences that are similar to the Cx_8_Cx_5_Cx_3_H RNA-binding motif: Cx_7_Cx_4_Cx_3_H and Cx_7_Cx_5_Cx_3_H [63] (Fig 1A, map (a)). Four regions, three of which are C-terminal to the zinc fingers, are predicted to be disordered by MobiDB [64]; the SPOT-Disorder algorithm [65] predicts that the entire region C terminal to the 2nd zinc finger is disordered (S1A Fig). ZC3H30 is found in all Kinetoplastid species sequenced so far, but most of the sequence similarity is concentrated around the two zinc finger domains (S1B Fig). The only other well-conserved sequence is a short motif, LR(I/V)(F/Y)DVR(P/G/T)(K/R) which starts at residue 397 of the *T*. *brucei* sequence. A BLASTp search did not find this motif in any other *T*. *brucei* protein and its significance is unknown.

We first wanted to check whether the results from the tethering screen were correct. We used cell lines constitutively expressing a chloramphenicol acetyl transferase (*CAT*) reporter gene, with or without 5 copies of ⎿-boxB hairpin between the *CAT* and the actin 3’ UTR (Fig 1A, RNA (c)). *CAT* mRNA without the boxB (B) sequence served as a negative control. We then integrated tetracycline-inducible plasmids encoding ZC3H30 with the λN peptide at the N terminus and a myc tag at the C terminus (⎿N-ZC3H30-myc, Fig 1A, protein (b)). Fig 1B shows that tethered ZC3H30 indeed reduces *CAT* mRNA and protein. The line expressing *CAT-boxB* and λN-ZC3H30-myc had very low levels of *CAT* mRNA and protein whether or not tetracyline was present (Fig 1B and 1C). The probable reason for this was that expression of ⎿N-ZC3H30-myc was detectable in the absence of tetracycline (Fig 1B). The line without boxB also showed a moderate supression of *CAT* mRNA, although the protein was not decreased. Although there was no obvious effect on cell growth, this might be a non-specific effect of ZC3H30 over-expression, but it was not investigated further.

From this experiment we concluded that tethered ZC3H30 may cause destruction of a bound mRNA, as predicted from the screening results. This does not, however, necessarily mean that ZC3H30 has the same activity when it is bound via its own RNA-binding domains.

### ZC3H30 deficiency impairs stress resistance

**Fig 2:**
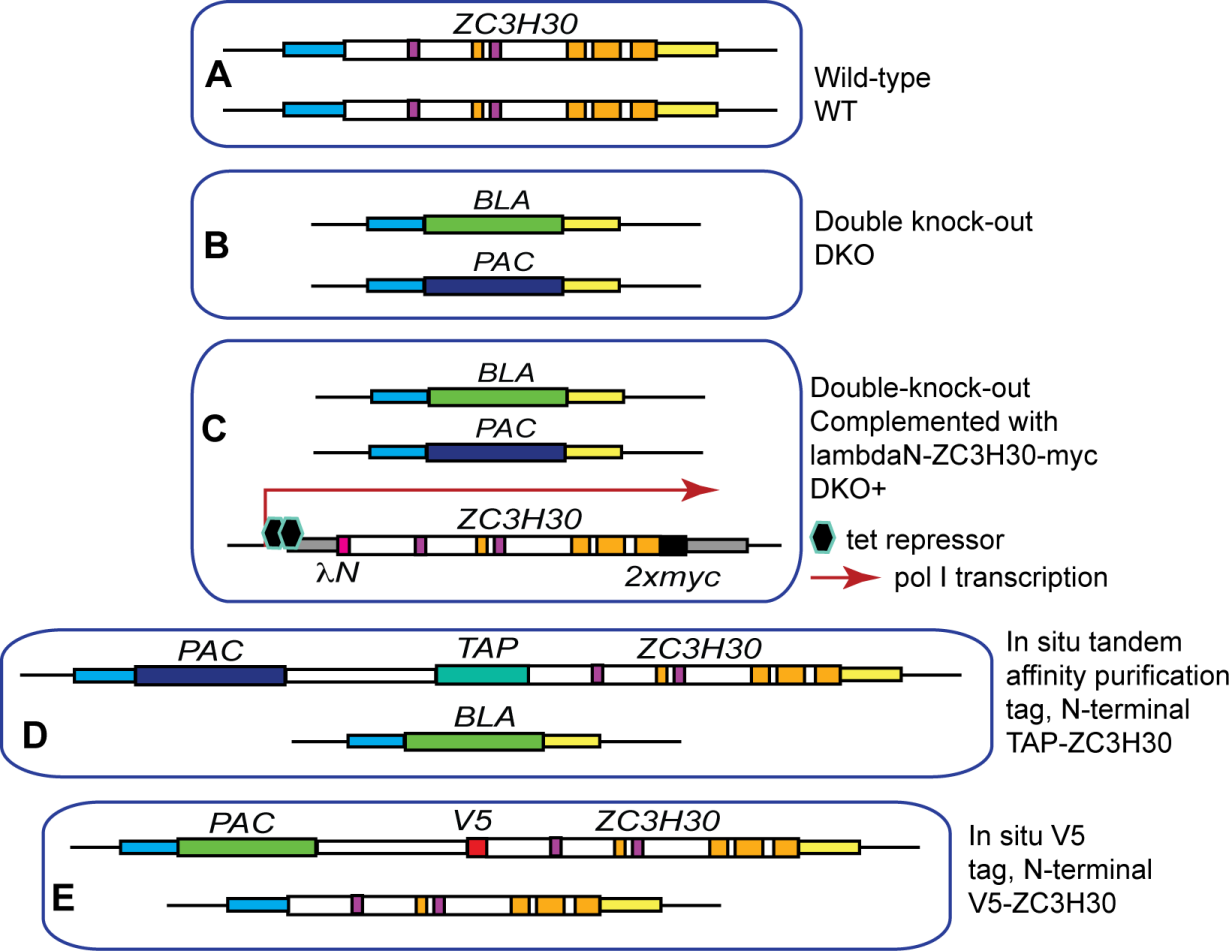
Cell lines used in this paper. Each frame represents a trypanosome nucleus with the relevant loci shown. The different panels A, B, C, D and E are labelled on the Figure and are described in the text. The diagrams are not to scale. Evidence for various lines is shown in S2 Fig and S3 Fig.

For our further investigations, we made a series of cell lines with altered ZC3H30 expression. We started with phleomycin-resistant wild-type (WT) cells expressing the *tet* repressor. The WT procyclic forms were also G418-resistant and expressed T7 polymerase, whereas the bloodstream forms were not G418 resistant. In both bloodstream and procyclic forms, both *ZC3H30* genes (Fig 2A) were replaced by selectable markers (DKO, Fig 2B, S2 Fig). Correct integration was confirmed by PCR (S2 Fig). Since clonal selection can result in accidental selection of additional mutations, and the presence of selectable markers might also affect the phenotype, lines complemented with tetracycline-inducible λN-ZC3H30-myc (DKO+, Fig 2C) served as controls. Procyclic and bloodstream-form DKO and DKO+ cells grew normally under standard conditions (S2B and S2C Fig). All tests of growth were done in cells that had been grown for three days without any selective drugs; but during this period tetracycline was included in DKO+ cultures.

We also made procyclic and bloodstream forms containing a single copy of *ZC3H30* bearing an N-terminal Tobacco-Etch-Virus-protease-cleavable tandem affinity purification (TAP) tag [62] (Fig 2D, S3A–S3C Fig), and procyclic- and bloodstream-form lines in which one copy of *ZC3H30* bore a V5 tag (Fig 2E, S3D Fig). Results from replicate experiments with this line indicated that procyclic forms have only 1.5 times more ZC3H30 than bloodstream forms (S3D Fig). Two V5-ZC3H30 bands were visible: this suggests that some of the ZC3H30 is post-translationally modified.

**Fig 3:**
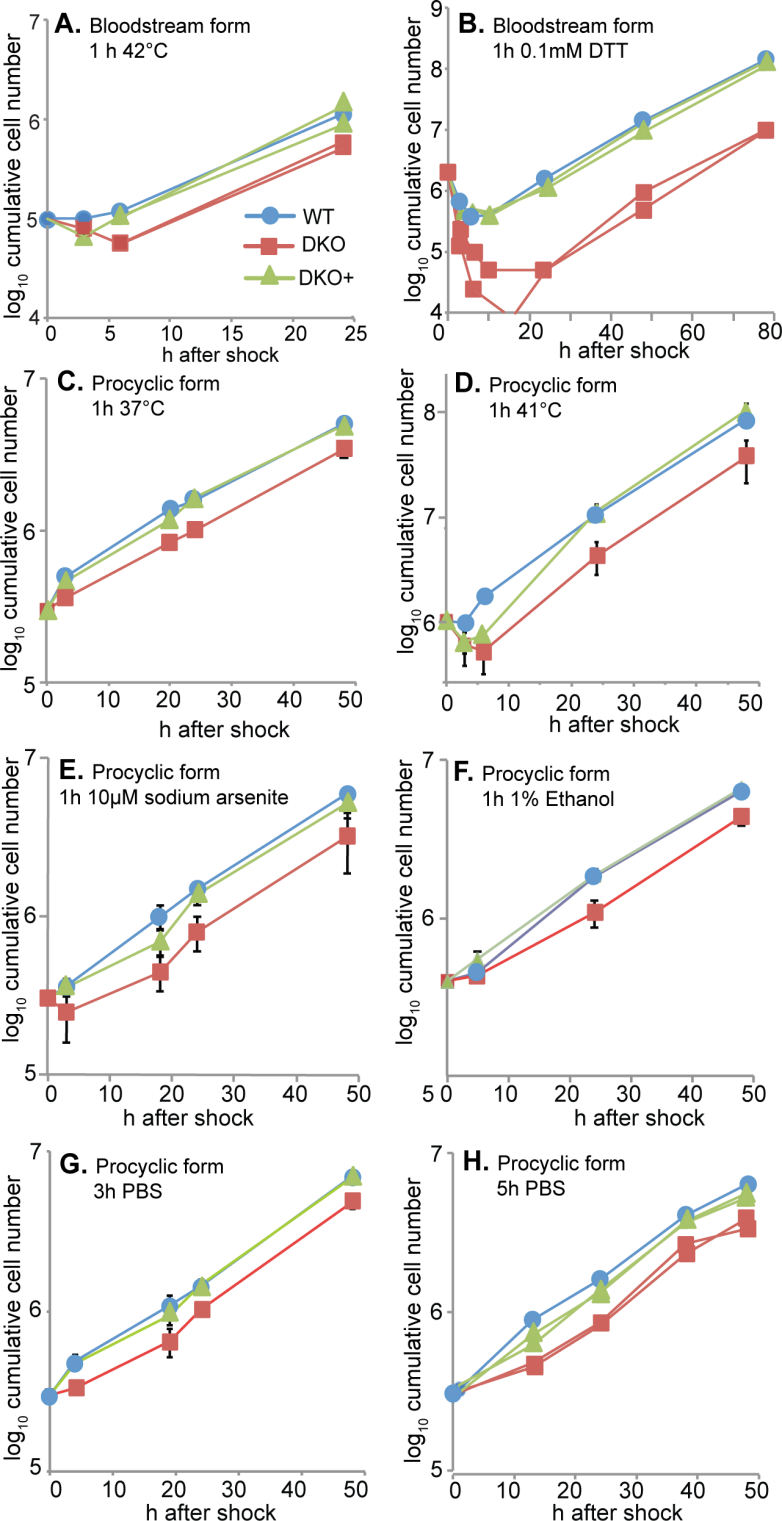
Trypanosomes lacking ZC3H30 are sensitive to stress. Each panel shows cell numbers after application of a physical or chemical stress. Cells were incubated without selective drugs and were all in exponential growth at the start of the experiments. The DKO+ cell cultures included tetracycline for three days prior to, and for all of the time during, the experiments. Stresses were applied, then the cells were centrifuged and resuspended in fresh medium. In some experiments cells were diluted as necessary. Additional stresses are shown in S4A–S4C Fig. In individual experiments, the replicates for DKO and DKO+ were for independent cultures, usually of different clones. When experiments were repeated, 2 clones were usually used. (A) Bloodstream forms, 1 h at 42°C. This is a single experiment and individual replicates are shown. For a similar experiment at 42°C see S4A Fig. (B) Bloodstream forms, 1 h with 0.1 mM DTT. This is a single experiment and individual replicates are shown. (C) Procyclic forms, 1 h at 37°C. This is a single experiment with 3x WT, 3x DKO (each in triplicate), and 3x DKO+. Standard deviations are not visible beneath the data points. (D) Procyclic forms, 1 h at 41°C. This is a single experiment with 1x WT, 4x DKO, and 3x DKO+. (E). Procyclic forms, 1 h with 10 μM sodium arsenite. This is from two experiments. Experiment 1 had time points 24h and 48h, with 1x WT, 3x DKO, and 3x DKO+. Experiment 2 had time points 3h, 18h, 24h and 48h, with 3x WT, 6x DKO, and 5x DKO+. For a similar experiment with 20μM sodium arsenite see S4B Fig. (F) Procyclic forms, 1 h at 1% ethanol. This is a single experiment, with 1x WT, 3x DKO, and 3x DKO+. For a similar experiment with 2% ethanol see S4C Fig. (G) Procyclic forms, 3 h in PBS. This is from two experiments. Experiment 1 had 2x WT, 3x DKO, and 2x DKO+. Experiment 2 had 1x DKO and 2x DKO+. (H) Procyclic forms, 5 h in PBS. This is a single experiment and individual replicates are shown.

Since *ZC3H30* DKO cells multiplied normally under standard culture conditions, we tested their response to adverse treatments, using DKO+ and WT cells as controls. Bloodstream forms were tested at 42°C and 41°C, while a heat shock temperature of 37°C was used for procyclics (Fig 3C). These temperatures might reasonably be expected to occur in the relevant animal hosts. In addition, we tested procyclic forms at 41°C (Fig 3D) because this temperature has been used in some previous publications. We also tested various other treatments: DTT, arsenite, ethanol, and for procyclic forms, starvation (incubation in buffered saline). In all cases except starvation, which was a 5-h treatment, cells were shocked for 1h then returned to normal growth conditions. The DKO bloodstream forms were deficient in the ability to recover after heat shock or DTT treatment (Fig 3A and 3B, S4A Fig). Similarly, procyclic DKO cells were more susceptible to arsenite (Fig 3E, S4B Fig), heat (Fig 3C and 3D), ethanol (Fig 3F, S4C Fig) and starvation (Fig 3G). Cells expressing only the TAP-tagged protein also grew like the wild type after heat shock (S3B Fig), confirming that the tagged protein was functional. During selection of cells expressing ZC3H30-myc, the cells lacking ZC3H30 seemed to survive rather longer than normal in our normal concentration of hygromycin. However when we tested susceptibility to aminoglycosides in more detail no difference was observed (S4D and S4E Fig).

### Protein synthesis after heat shock of cells lacking ZC3H30

**Fig 4:**
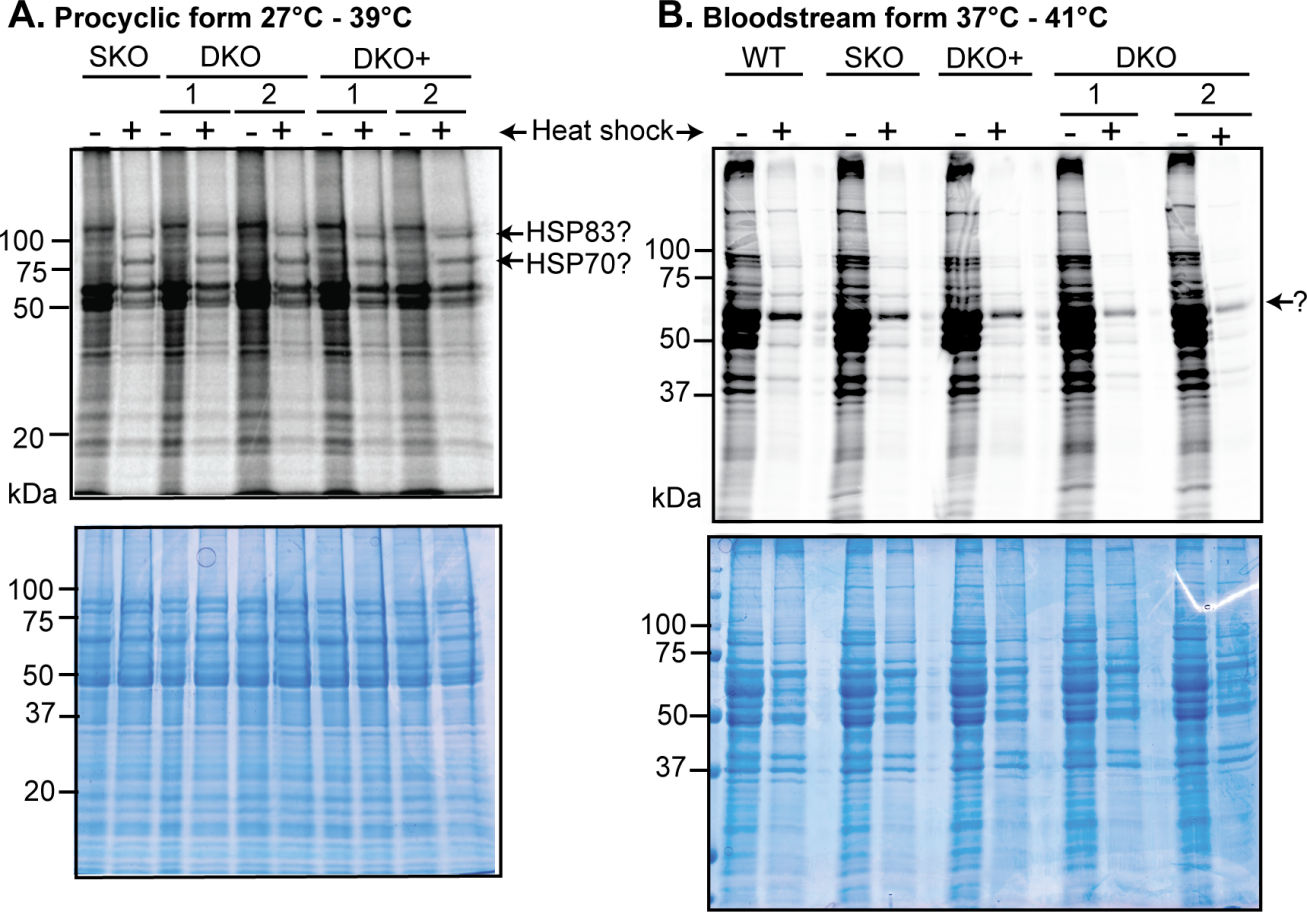
Protein synthesis. Trypanosomes were subjected to heat shock for 30 min in medium lacking methionine, then [^35^S] methionine was added for 30 min. Cells were washed once then the pellets were boiled in sample buffer and subjected to SDS-PAGE followed by autoradiography. (A) shows results for procyclic forms: the positions at which HSP83 and HSP70 may migrate are indicated. (B) shows results from bloodstream forms. The identity of the prominent band (marked ?) is not known but from its size, it might be variant surface glycoprotein.

For about 30–60 min after heat shock, mRNAs encoding chaperones continue translation, while other mRNAs lose translation and are degraded [19]. We therefore looked to see whether DKO cells retained this heat shock response. We subjected procyclic trypanosomes to a 39°C shock, then pulsed them with [^35^S]-methionine to measure protein synthesis. 39°C was chosen because we had previously characterised transcriptomes of WT procyclic forms at this temperature [25]. The control shocked SKO cells had reduced protein synthesis (Fig 4A), with two clear labelled bands at 70 kDa and 90 KDa (Fig 4A); these may correspond to HSP70 and HSP83 (the trypanosome equivalent of HSP90). The DKO cells responded similarly, suggesting that the heat shock response was unimpaired. Preferential synthesis of HSP70 and HSP83 was not so evident in bloodstream forms shocked at 41°C (Fig 4A), but quantitation again showed no major differences in cells lacking ZC3H30. Preliminary polysome profiling results showed that most ZC3H30 is not associated with polysomes or ribosomal subunits (S5A and S5B Fig). We have however previously shown that this result is expected for an mRNA-binding protein unless it binds to multiple sites on many mRNAs [66]. Unfortunately we could not measure HSP70 protein synthesis specifically because we could not find a suitable antibody, so we could not assign the radioactive bands with any certainty.

After heat shock, ZC3H11 is required for the heat shock response, and translation of the *ZC3H11* mRNA is enhanced by heat shock [26, 27]. We therefore wondered whether the lack of ZC3H30 resulted in low ZC3H11 expression, which in turn would make the cells vulnerable to stress. This idea was incorrect. ZC3H11 induction was indistinguishable in DKO, DKO+ and WT cells (S4F and S4G Fig).

### mRNA levels in cells lacking ZC3H30

**Fig 5:**
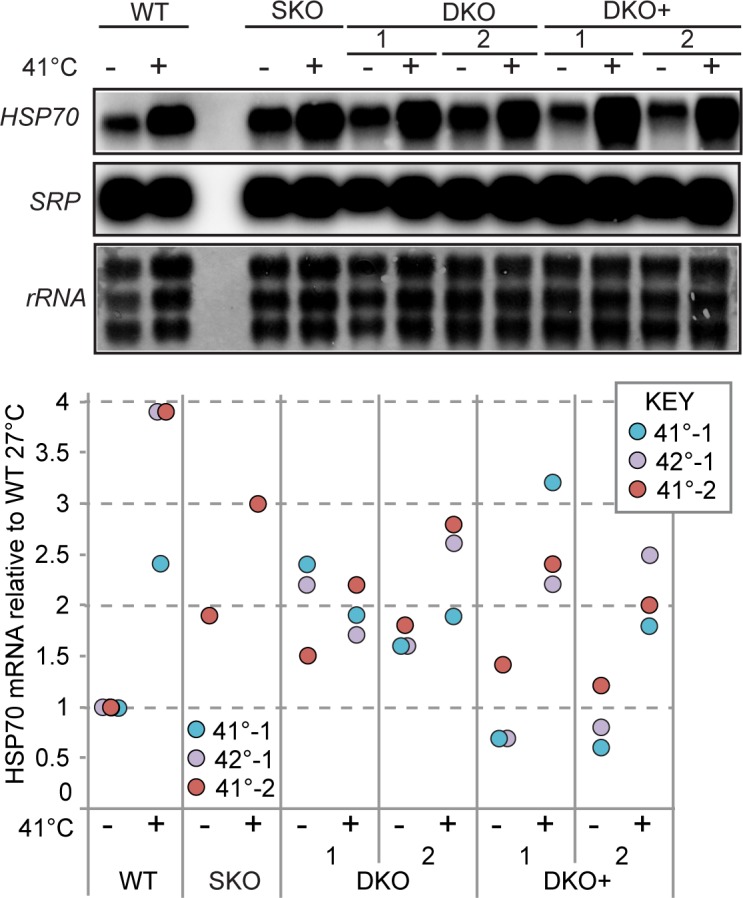
*HSP70* mRNA in bloodstream forms lacking ZC3H30. RNA was isolated from bloodstream-form trypanosomes with and without a 1h 41°C heat shock. *HSP70* mRNA ws detected with a radioactive probe and the signal was quantitated relative to the *SRP* RNA. The lower panel shows quantitation for three biological replicates, as indicated on the graph. The signal for WT cells at 27°C was set at 1.0. Additional replicates are shown in S4F and S4G Fig.

Concurrently with the analyses of translation shown in Fig 4, we examined *HSP70* mRNA levels (Tb927.11.11330) in the different cell lines. Preliminary experiments with procyclic forms (S4H Fig) suggested that relative to WT, *HSP70* mRNA was elevated in the DKO cells grown at 27°C, but the same was not true for the DKO+ cells. After a 39°C heat shock, the *HSP70* mRNA levels in all three cell types were similar. Bloodstream-form *ZC3H30* DKO trypanosomes grown at their normal temperature of 37°C also had roughly 2-fold more *HSP70* mRNA than both WT and DKO+ cells (Fig 5, S4I Fig). However a single test of the SKO line suggested that it, too, had more *HSP70* mRNA than normal (Fig 5).

**Fig 6:**
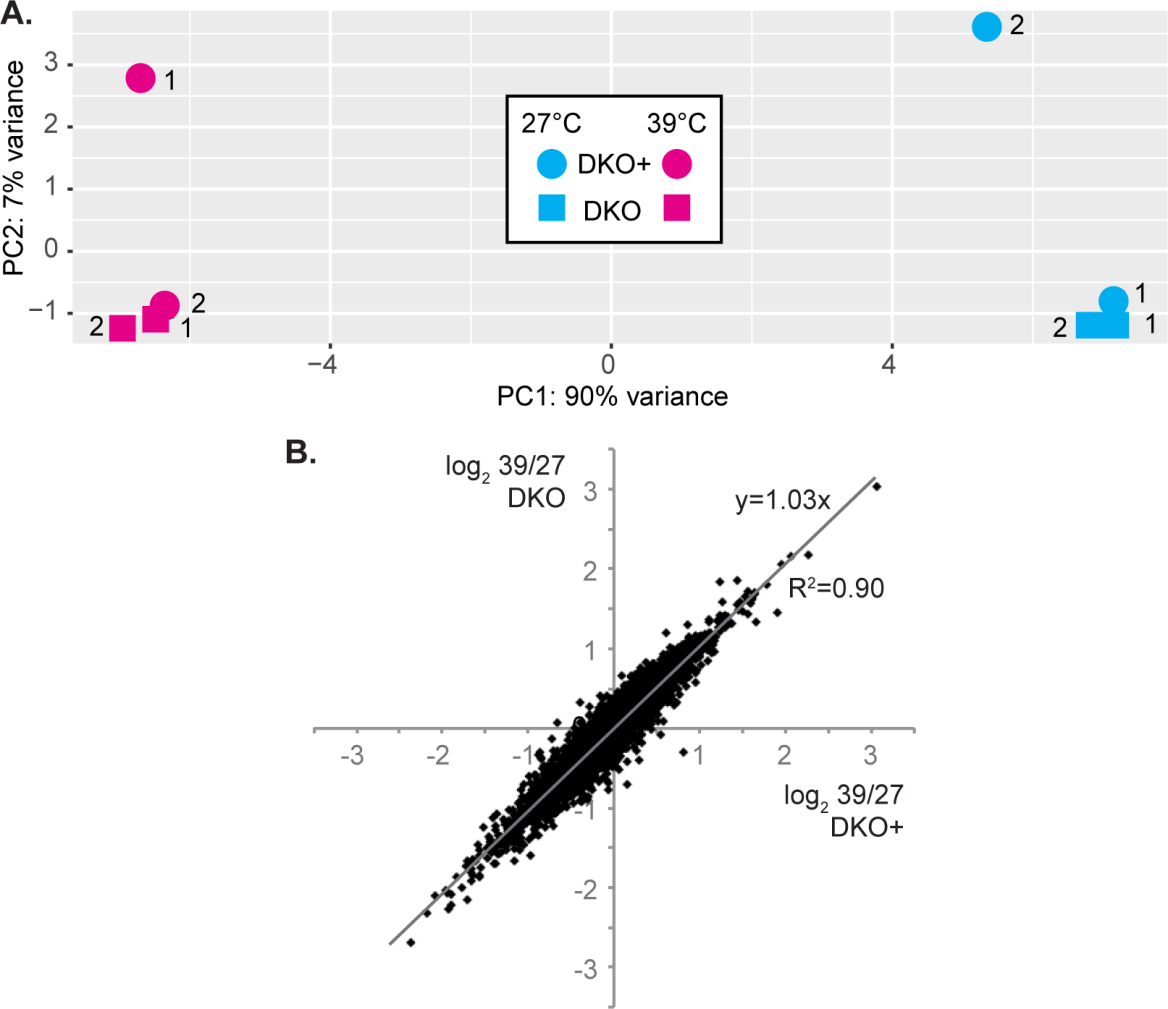
Transcriptomes of ZC3H30 DKO and DKO+ procyclic forms are similar. (A) Principal component analysis for *ZC3H30* DKO and DKO+ cells. The key is on the Figure. Note that 90% of the variance is on the x-axis. The two replicates are numbered. (B) Effect of heat shock on transcriptomes. Each point represents one gene. The log_2_ fold change for DKO+ cells is on the x-axis and the log_2_ fold change for DKO cells is on the y-axis.

To find out whether any other mRNAs were affected by loss of ZC3H30, we examined the transcriptomes of procyclic DKO and DKO+ cells at 27°C and after 1h at 39°C. These conditions were chosen because we had already characterised transcriptomes of WT procyclic forms after the same treatment, using the same RNA preparation and sequencing methods [25]. The transcriptomes from the DKO and DKO+ cells were highly reproducible (S6A and S6B Fig), but surprisingly, apart from *ZC3H30*, there were no significant differences between DKO and DKO+ cells (Fig 6A; S6C and S6D Fig, S2 Table sheets 1 and 2). The heat shock responses in DKO and DKO+ cells were also almost indistiguishable and similar to the changes seen previously in WT cells (Fig 6B, S2 Table sheets 1 and 2). The increased mRNAs were enriched in the functions "chaperone" (Fisher Padj 3 x 10^−8^), "RNA-binding protein" (0.03) and "ESAG" (0.03). Decreased mRNAs encoded, in particular, proteins of the classes "mitochondrial electron transport" (1 x 10^−4^), "mitochondrial pathway" (0.01) and "nucleotides" (9 x 10^−3^). In disagreement with the Northern blot result, *HSP70* mRNA abundances were almost identical in the two procyclic cell lines at 27°C and were induced to similar extents at 39°C. The transcriptome results therefore gave no clue as to why the ZC3H30 DKO procyclic forms were stress-susceptible.

### ZC3H30 RNA interactions

We next wished to find out whether ZC3H30 binds to a specific subset of mRNAs. We therefore subjected TAP-ZC3H30 from procyclic trypanosomes to tandem affinity purification and characterised the bound RNAs in duplicate preparations by RNASeq. The two bound fractions were reasonably similar (correlation coefficient 0.87) (S3 Table). 96 mRNAs were more than 2-fold more abundant in both bound fractions than in two total RNA preparations (from DKO+ cells) and one unbound fraction (S3 Table, sheet 3). Of the 96 "bound" mRNAs, 47 are reproducibly at least 2x more abundant in bloodstream forms than in procyclic forms, and 9 are more abundant in procyclic forms. In contrast, for the 1335 "unbound" mRNAs (ratio less than 1), 38 are more abundant in bloodstream forms and 390 more abundant in procyclic forms (S3 Table, sheet 3). This tendency is also apparent on a scatter plot of the whole dataset (S7C Fig). A comparison of the transcriptome results (DKO relative to DKO+) with those for RNA binding revealed no correlation. Any effect of ZC3H30 on bound mRNAs would therefore have to be on translation or localisation, rather than abundance.

**Fig 7:**
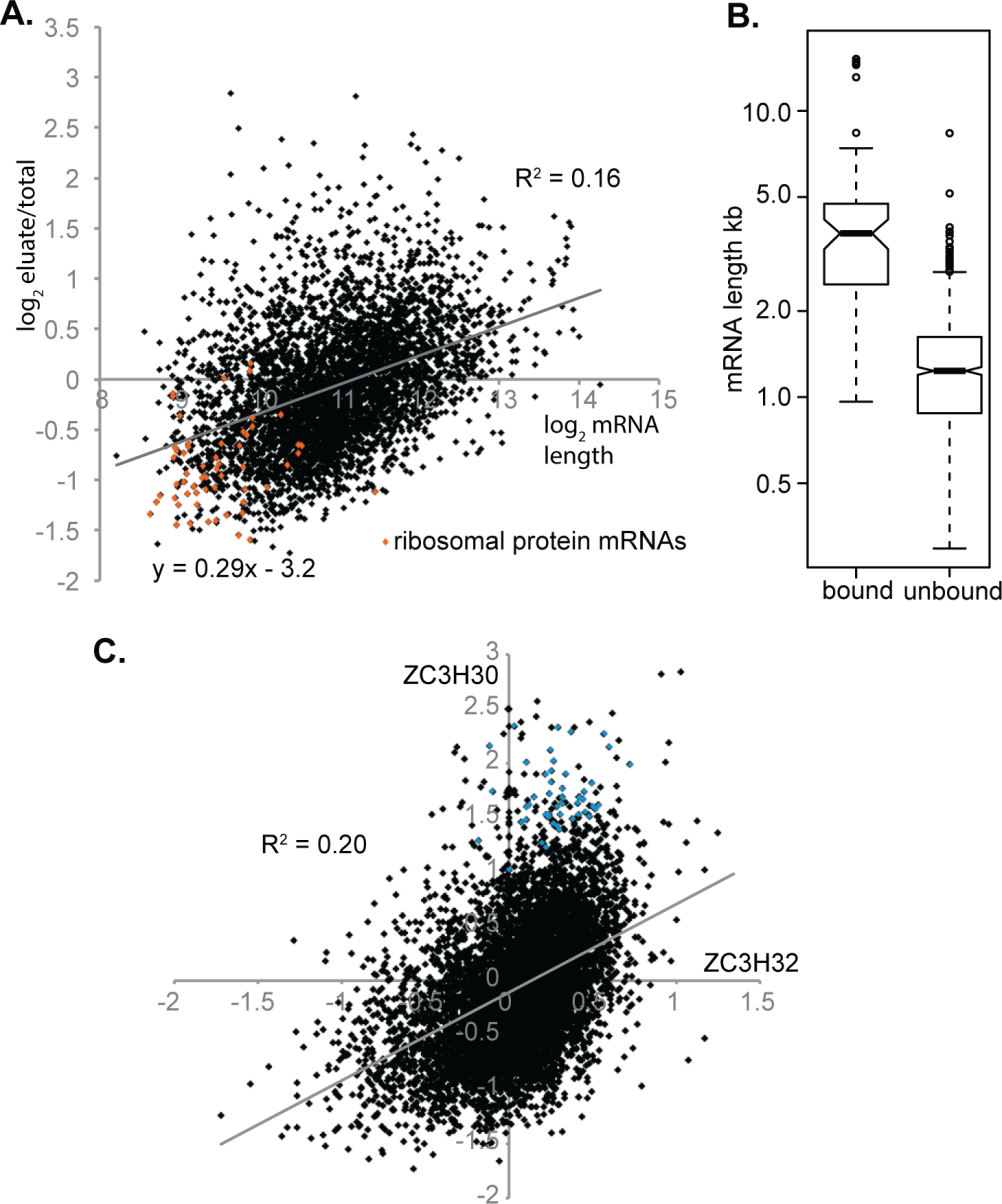
The association of mRNAs with ZC3H30 is weakly correlated with mRNA length. (A) The average enrichment of each mRNA (eluate RPM / input RPM) was plotted (y-axis) relative to the annotated mRNA length (x-axis). Both are on a log_2_ scale. Ribosomal protein mRNAs are in orange. Note that some annotated mRNA lengths are too short, usually due to underestimation of 3'-untranslated regions. (B) The lengths of mRNAs that were reproducibly enriched in the ZC3H30 pull-down (all ratios >2) and those that reproducibly were not enriched (all ratios less than 1) are shown as box plots. For gene lists see S3 Table, sheet 3. (C) Relationship between RNA binding by ZC3H30 and ZC3H32. The x-axis shows binding to ZC3H32 (average of three bound/eluate) and the y-axis shows the average of bound/DKO+ (total) for ZC3H30, both on a log_2_ scale. The cyan spots represent mRNAs that were reproducibly enriched in the ZC3H30 pull-down and for which the enrichment ratio was at least 2-fold higher with ZC3H30 than with ZC3H32.

When we examined the complete dataset, we observed a weak correlation between the length of mRNAs and enrichment in the bound fraction (Fig 7A and 7B). The length alone seemed to be important since there was almost no relationship if the coding region and 3'-untranslated region were considered separately (S7A and S7B Fig). We therefore suspected that binding to ZC3H30 (or perhaps just to the beads) might have low sequence specificity. Notably, when we plotted developmental regulation against mRNA length we discovered that mRNAs that are more abundant in procyclic forms tend to be shorter than those that are more abundant in bloodstream forms (S7D and S7E Fig). The preference of ZC3H30 to bind bloodstream-specific mRNAs may, therefore, be a side-effect of the fact that these mRNAs tend to be longer.

For mRNA-binding proteins that have strong mRNA-binding specificity, such as RBP10 [10] and ZC3H11 [26], there is no relationship between mRNA binding and length. In contrast, the CCCH zinc finger protein ZC3H32, like ZC3H30, showed binding that increased with mRNA length—although for ZC3H32 the correlation was much stronger (R^2^ = 0.44) [67]. We therefore compared the two sets of results (S3 Table, sheet 3). We found 49 ZC3H30-bound mRNAs that were at least two-fold more enriched in the ZC3H30 pull-down than in the ZC3H32 pull-down (Fig 7C). In the list of gene products for enriched mRNAs, citric acid cycle enzymes (aconitase, fumarate hydratase, glutamate dehydrogenase, isocitrate dehydrogenase), and enzymes of glucose metabolism (fructose bisphosphate aldolase, phosphofructokinase, pyruvate kinase and pyruvate phosphate dikinase) were over-represented, as were RNA binding proteins (DRBD3, DRBD18, PUF9, TRRM3 and ZC3H28) (S3 Table, sheet 3). There is no obvious connection between these gene products and stress susceptibility. It is also not clear whether the selection of these particular mRNAs is meaningful: 49 genes is less than 1% of all genes considered, so it is possible that this list represents random noise, rather than specific selection by ZC3H30. If the selection is real, changes in expression of the encoded proteins might influence stress resistance indirectly, for example via alterations in metabolism.

### ZC3H30 interacts with a stress granule protein

**Fig 8:**
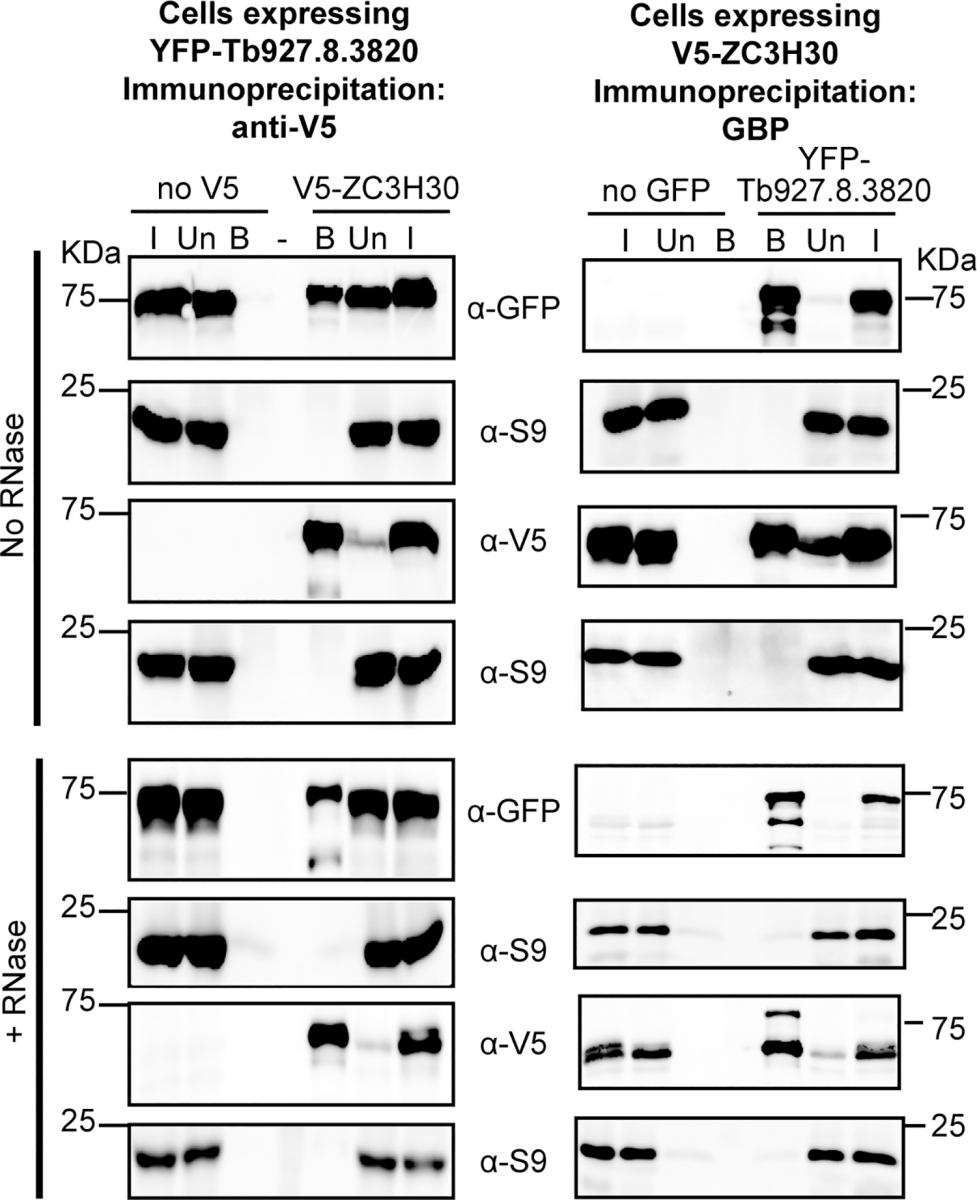
ZC3H30 interacts with the protein encoded by Tb927.8.3820. Proteins were immunoprecipitated from cell lines expressing V5-ZC3H30 and/or YFP-927.8.3820, then analysed by Western blotting with the indicated antibodies. Ribosomal protein S9 served as a negative control. I = Input; Un = Unbound; B = Bound. Left panel: immunoprecipitation with anti-V5 (for V5-ZC3H30); right panel: immunoprecipitation with anti-GFP (for YFP-927.8.3820). For RNase treatment, the lysis and wash buffers contained 200 μg/mL of RNase A. Both anti-V5 and anti-GFP antibodies detected degradation products in addition to the full-length fusion proteins (S8 Fig); here, only the full-length bands are shown.

Finally, we searched for protein-protein interactions. Analysis of triplicate affinity-purified preparations, made both with and without RNase, confirmed strong enrichment of ZC3H30 (S4 Table). The experiments revealed a single specific ZC3H30 interaction partner encoded by Tb927.8.3820. Using procyclic *in-situ-*tag cell lines expressing YFP-Tb927.8.3820 [24] and/or V5-ZC3H30, we confirmed the interaction by reciprocal immunoprecipitation (Fig 8). Immunoprecipitation with anti-V5 pulled down YFP-Tb927.8.3820 only if V5-ZC3H30 was present, and ribosomal protein S9 was not co-immunoprecipitated (left panel). Similarly, immunoprecipitation with anti-GFP pulled down V5-ZC3H30 only if YFP-Tb927.8.3820 was present (right panel). As in the tandem affinity purification, the interaction was not prevented by inclusion of RNase.

**Fig 9:**
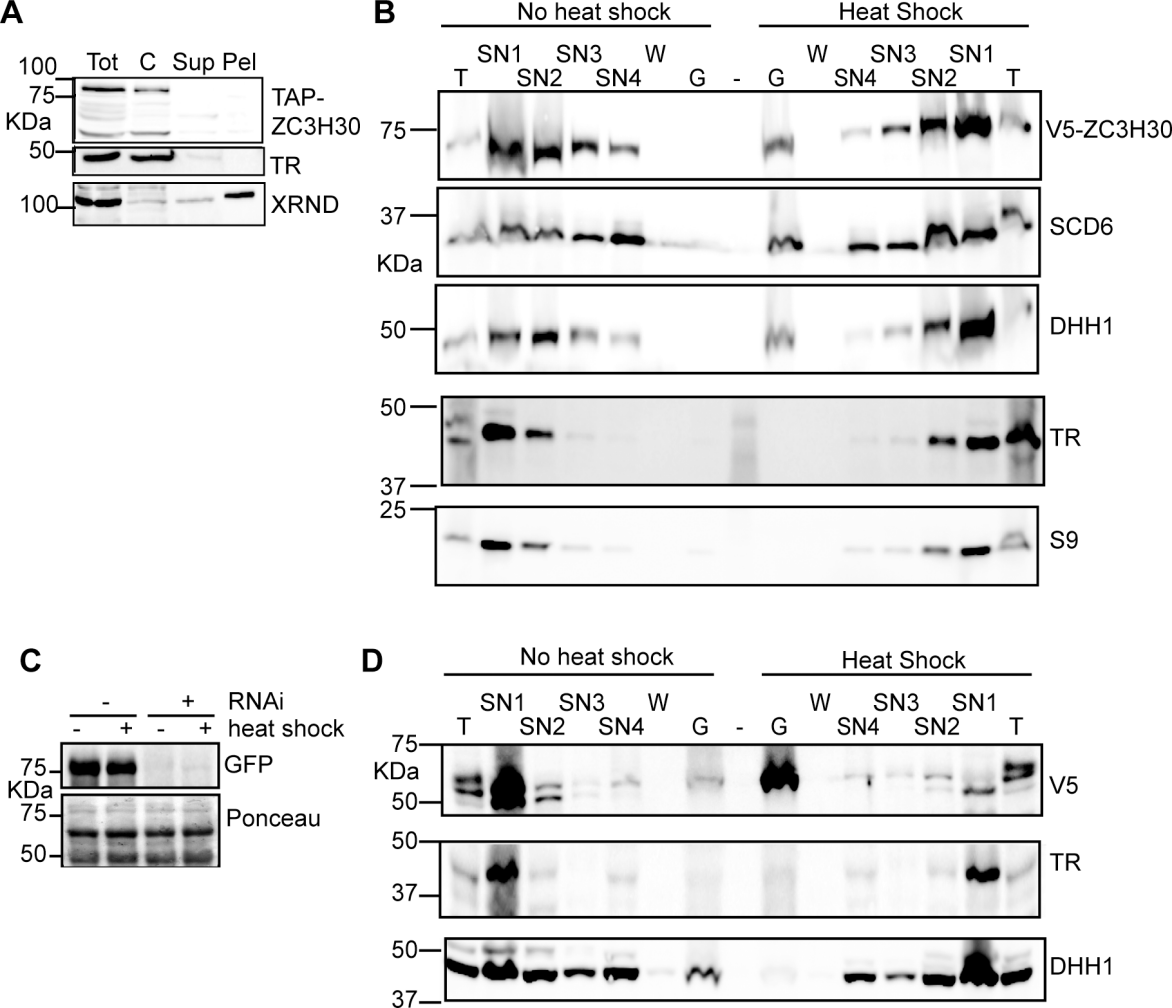
ZC3H30 is in a granule fraction after heat shock. A) In procyclic trypanosomes grown at 27°C, ZC3H30 is in the cytosolic fraction. Trypanosomes were disrupted using silicon carbide, and fractionated by centrifugation. 5 x 10^6^ cell-equivalents were loaded on each lane of an SDS-PAGE gel and the proteins were detected by Western blotting. Tot: total lysate; C: cytosol: Sup: detergent-soluble organellar proteins; Pel: IGEPAL-insoluble pellet. TR is trypanothione reductase, a cytosolic protein, and XRND is in the nucleus. (B) ZC3H30 moves to the granule fraction after heat shock. Trypanosomes expressing V5-ZC3H30, with or without a 1h heat shock at 41°C, were fractionated into various soluble and granular fractions, using the cytoskeleton as a molecular sieve to trap stress granules. T: Unfractionated total cell lysate; SN1-3: soluble supernatants; SN4: molecules trapped inside cytoskeletons but not sedimented at 20000g (10min); G: sedimentable granules. Proteins were detected from equal proportions of each fraction. DHH1 and SCD6 are stress granule markers and trypanothione reductase (TR) is in the soluble fraction. (C) Loss of GFP-Tb927.8.3820 after 24h RNAi induction in procyclic forms (control for panel (D)). The Ponceau protein stain serves as a loading control. (D) ZC3H30 is in the granule fraction in heat-shocked Tb927.8.3820-depleted cells. As in (B), but using cells with RNAi against Tb927.8.3820 (see (C)).

Both ZC3H30 and the Tb927.8.3820 protein were previously found in purified procyclic form starvation granules [24], and GFP-tagged ZC3H30 exhibits a cytosolic, granular pattern in procyclic forms that have been incubated in PBS (see http://tryptag.org/?id=Tb927.10.1540). We wondered whether Tb927.8.3820 was needed for ZC3H30 recruitment to stress granules. By immunoflourescence, the signal from V5-ZC3H30 was barely distinguishable from background, but cell fractionation experiments confirmed that V5-ZC3H30 is in the cytoplasm (Fig 9A) and enriched in the granular fraction after heat shock [24, 25] (Fig 9B). Although formation of aggregates without RNA cannot be ruled out, this result suggests that ZC3H30 is indeed associated with RNA-protein stress granules. Then, using the cell line expressing YFP-Tb927.8.3820 and V5-ZC3H30, we depleted Tb927.8.3820 by RNA interference (Fig 9C, S8A Fig). Results from a high-throughput RNAi screen had suggested that Tb927.8.3820 is important for cell growth in all tested life-cycle stages, but we detected no effect of RNAi in procyclic forms, even after heat shock or arsenite treatment (S8A Fig). Depletion of Tb927.8.3820 also did not affect partitioning of ZC3H30 into the granule fraction (Fig 9C). It is, however, possible that the amount of Tb927.8.3820 protein that remained after RNAi was sufficient for normal function. Preliminary tests revealed no strong effects of stress on the abundances of either ZC3H30 or Tb927.8.3820 proteins in procyclic forms (S8B Fig).

## Discussion

The results from this study show that zinc finger RNA binding protein ZC3H30 is not essential for growth and proliferation of bloodstream-form or procyclic-form trypanosomes under normal culture conditions. However, loss of ZC3H30 results in the cells being more susceptible than the wild type to many stresses. These changes were lost when expression of ZC3H30 was restored. ZC3H30 associates with the stress granule protein encoded by Tb927.8.3820, but RNAi targeting Tb927.8.3820 did not affect the association of ZC3H30 with the granule fraction after heat shock. The transcriptomes of procyclic forms lacking ZC3H30 were similar to those of cells in which ZC3H30 was re-expressed, so if ZC3H30 does affect the mRNAs to which it is naturally bound (rather than tethered), it must influence translation or localisation, rather than mRNA abundance.

ZC3H30 is associated with mRNA, and was found in the granule fraction. It is therefore possible that ZC3H30 is involved in the sequestration of mRNAs in granules in response to stress. Conversely, it is possible that ZC3H30 moves to the granules because it is associated with mRNAs. Either hypothesis would be consistent with the relative lack of RNA-binding specificity that we observed. There was no correlation between ZC3H30 binding and the percentage of the mRNA that was found in granules at 41°C [25], with one notable exception: ZC3H30 was not bound to most ribosomal protein mRNAs and these are also almost completely excluded from granules. However, ZC3H30 was also not much bound to many other mRNAs which are indeed found in stress granules.

The stress susceptibility of cells lacking ZC3H30 suggests that they would have a selective disadvantage in the wild. In both mammalian and tsetse fly hosts, trypanosomes are subject to temperature fluctuations [68–71] and stresses from immune reactions. It is also possible that dense trypanosome populations in tsetse experience decreased nutrient availability. It would be interesting to investigate whether ZC3H30 is also required for stress resistance and/or intracellular infection in *Trypanosoma cruzi* or *Leishmania*. Conservation of ZC3H30 in both monogenetic and digenetic parasites suggests that it may be particularly important within arthropod or reptile hosts, where temperature fluctuations are much wider than within mammals.

## Supporting information

S1 Fig Sequence and structure of ZC3H30. A. SPOT disorder plot for ZC3H30. B. Alignment with ZC3H30 from other kinetoplastids. Sequences are: TcIL3000_10_1340.1—*Trypanosoma congolense*; Baya_167_0060—*Blechomonas ayalai*; Lsey_0021_0550–1—*Leptomonas seymouri*; TvY486_1001540—*Trypanosoma vivax*; CFAC1_130011500—*Crithidia fasciculata*; LmjF.21.0770—*Leishmania major*; EMOLV88_210012500—*Endotrypanum monterogeii*; Tc_MARK_1002. -*Trypanosoma cruzi*. The Alignment was done using MegAlign and a key is on page 2.
(PDF)

S2 Fig Elimination of the *ZC3H30* open reading frame (ORF) in procyclic-form (PC) and bloodstream-form (BS) trypanosomes. A) Schematic representation of *ZC3H30* alleles (a), the resistance cassettes used to replace them by homologous recombination (b,c) and the indiucible lambdaN-ZC3H30-myc cassette (d). Only the *ZC3H30* coding region is to scale. Primer locations are indicated by small arrows and sizes of PCR products are also shown. B) Knockout in procyclic forms. (a) Ethidium-bromide-stained agarose gel pictures for PCR products shown in (A); (b) Western blot showing expression of lambdaN-ZC3H30-myc in DKO+ lines with and without tetracycline; (c) growth of three lines, each measured in duplicate, with cumulative counts shown as mean ± standard deviation. C) As (B), but for the knock-out in bloodstream forms. "SKO" is the single knock-out line with only puromycin resistance. For unknown reasons, then *PAC* amplification failed in all but one preparation. D) Repeat PCRs done for the procyclic cell lines and an independent knockout in bloodstream forms.
(PDF)

S3 Fig Cell line for tandem affinity purification. A) (a) Schematic representation of *ZC3H30* alleles; (b) the *in situ* TAP-tagged allele; and (c) the integrated blasticidin resistance cassette. Only the *ZC3H30* coding region is to scale. Primer locations are indicated by small arrows and sizes of PCR products are indicated. B) Ethidium-bromide-stained agarose gel pictures for PCR products shown in (A). In the cell line expressing only TAP-ZC3H30, the PCR product for F1-Rcds should be 2.6 kb but no band was obtained. Presumably the PCR conditions were inappropriate for this particular product. Successful expression was however seen both by Western blotting (Fig 6A) and mass spectrometry (S4 Table). C) Growth of cells expressing only TAP-ZC3H30 after a heat shock (1h, 41°C) compared with WT. D) Expression of V5-ZC3H30 in cell lines with one *in situ*-tagged copy and one Wt copy of the gene. Western blots were incubated with antobodies detecting the indicated proteins.
(PDF)

S4 Fig Effect of various stresses on cells lacking ZC3H30. A. Growth of bloodstream forms after 1h at 41°C. This was a single experiment, individual data points are shown. B. Growth of procyclic forms after 1h in 20**μ**M sodium arsenite. This is combined data from two experiments. Experiment 1 had time points 3h, 6h, 24h and 48h, with 1x WT, 1x DKO, and 2x DKO+. Experiment 2 had time points 3h, 24h and 48h, with 2x WT, 4x DKO, and 2x DKO+. C. Growth of procyclic forms after 1h in 2% ethanol. This is a combination of two independent experiments. Experiment 1 had time points 3h, 24h and 48h, with 2x WT, 6x DKO, and 4x DKO+. Experiment 2 had time points 9h, and 28h, with 1x WT, 3x DKO, and 3x DKO+. For the graph, the data from the 24h and 28h have been put together and placed at 26h. D, E) Dose-response curves for procyclic forms grown with hygromycin (D), and bloodstream forms grown with G418 (E). F, G) Expression of ZC3H11 in bloodstream (F) and procyclic (G) forms after heat shock, detected by Western blotting. Using total cell lysates, ZC3H11 is normally obscured by background from tubulin. Cells are therefore fractionated to remove triton-insoluble cytoskeletons, before SDS-PAGE [27]. The background bands on the Western blots originate from residual cytoskeletal proteins and can be used as the loading control ("Load"). H) Levels of *HSP70* mRNA in procyclic forms with and without heat shock. Quantitation of the blot signals relative to WT is shown below. The experiment was not repeated because we instead subjected RNA to RNASeq and could not detect any effect of ZC3H30 on *HSP70* mRNA. I) Levels of *HSP70* mRNA in bloodstream forms with and without heat shock. These blots are quantitated in Fig 5.
(PDF)

S5 Fig ZC3H30 is not associated with polysomes in procyclic forms. A) Extracts from cells expressing *in situ* V5-tagged ZC3H30 were fractionated on sucrose gradients; the upper panel shows absorbance at 254 nm (arbitrary units) and the lower panels are Western blots probed with antibodies as indicated. S9 is ribosomal protein S9 and TR is trypanothione reductase; relevant marker molecular weights (in kDa) are also indicated. The least dense fractions are on the left. B) As (A) but after a 39°C heat shock; TxNPx is tryparedoxin peroxidase.
(PDF)

S6 Fig Correlation of RPM for all unique genes, comparing replicates and chosen DKO/DKO+ pairs. **Each spot represents a gene.** Data are in S2 Table. Panels A, B, C and D are comparisons of different paired datasets as indicated on the "x" and "y" axes.
(PDF)

S7 Fig RNAs associated with TAP-ZC3H30. In each plot, each spot represents a single unique gene. A. There was no correlation between TAP-ZC3H30 binding and mRNA coding region (CDS) length. On the y-axis, we have plotted (on a log_2_ scale) the average RPM from both eluates divided by the average RPM from DKO+ cells. In all graphs, regression lines and correlation coefficients were calculated in Microsoft Excel. B) As (A), except that this is the relationship between binding and mRNA 3'-UTR length. C) Relationship between binding and developmental regulation of mRNA abundance [44]. The y-axis shows the log_2_-transformed ratio between procylic- and bloodstream-form expression. D) Relationship between mRNA length and developmental regulation of mRNA abundance. E) Relationship between mRNA length and developmental regulation of total ribosome footprints [42]. F) Relationship between binding to ZC3H30 and developmental regulation of total ribosome footprints [42].
(TIF)

S8 Fig RNA targeting Tb927.8.3820 does not affect growth or stress resistance of procyclic forms. A) Growth of cells with RNAi under normal conditions, or after arsenite or a mild heat shock. The RNAi was done in cells expressing YFP-927.8.3820 and the equivalent Western blot is shown beneath the left-hand plot. The top band has the expected size but we usually saw a second band, which might be a degradation product. B) Effects of various stresses on the abundances of V5-ZC3H30 and YFP-8.3820 in procyclic forms. Sample blots are shown and quantifications for 2–4 measurements of V5-ZC3H30 are shown on the right. There was also no significant change in YFP-8.382 after stress. A second band of V5-ZC3H30 was routinely seen. Arrows indicate the migration of the full-length proteins.
(PDF)

S1 Table Plasmids, oligonucleotides and selective drug concentrations. (XLSX)

S2 Table Transcriptomes of trypanosomes lacking ZC3H30. For a detailed legend see the top sheet of the Table.
(XLS)

S3 Table mRNAs that copurify with TAP-ZC3H30. For a detailed legend see the top sheet of the Table.
(XLSX)

S4 Table Proteins that co-purify with TAP-ZC3H30. For a detailed legend see the top sheet of the Table.
(XLS)
