# Supplementary material for: Stress susceptibility in Trypanosoma brucei lacking the RNA-binding protein ZC3H30
Source: PLoS Negl Trop Dis. 2018 Oct 1;12(10):e0006835. doi: 10.1371/journal.pntd.0006835 (PMC6181440; doi:10.1371/journal.pntd.0006835)
Supplement: S1 Fig — A. SPOT disorder plot for ZC3H30. B. Alignment with ZC3H30 from other kinetoplastids. Sequences are: TcIL3000_10_1340.1—Trypanosoma congolense; Baya_167_0060—Blechomonas ayalai; Lsey_0021_0550–1—Leptomonas seymouri; TvY486_1001540—Trypanosoma vivax; CFAC1_130011500—Crithidia fasciculata; LmjF.21.0770—Leishmania major; EMOLV88_210012500—Endotrypanum monterogeii; Tc_MARK_1002. -Trypanosoma cruzi. The Alignment was done using MegAlign and a key is on page 2. (PDF) [file pntd.0006835.s001.pdf]

A

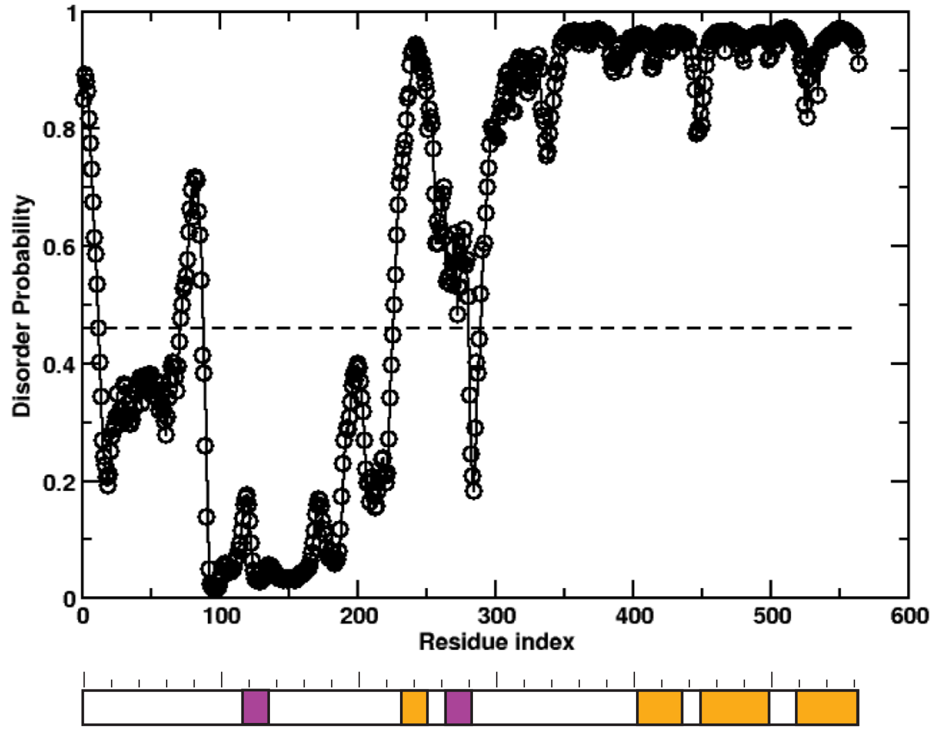

Disorder Probability
0
0.2
0.4
0.6
0.8
1
0
100
200
300
400
500
600
Residue index

B

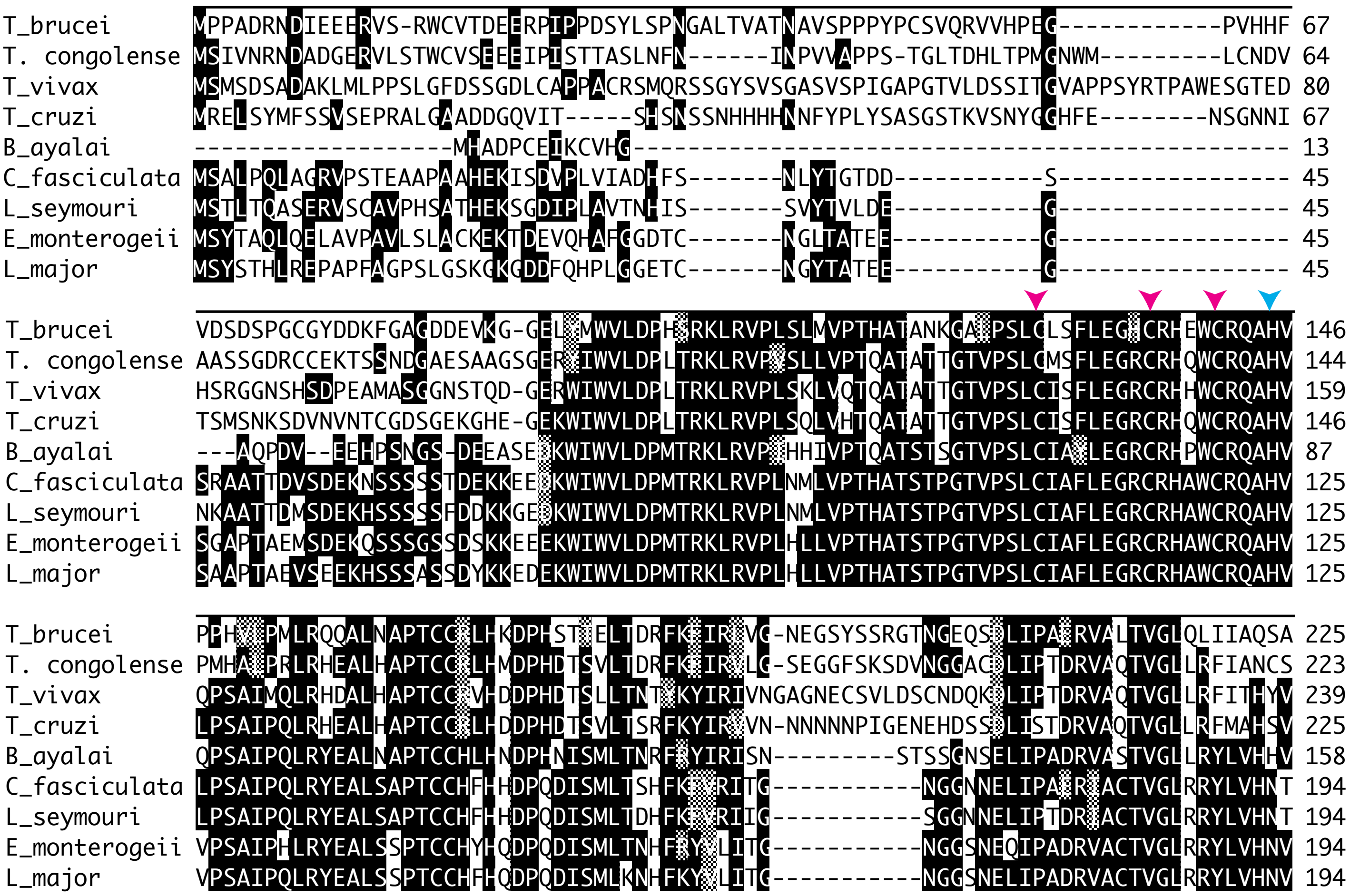

T_brucei MPPADRNDIEEERVS-RWCVTDEERPIPPDSYLSPNGALTVATNAVSPPPYPCSVQRVVHPEG------------PVHHF 67
T. congolense MSIVNRNDADGERVLSTWCVSEEEIPISTTASLNFN------INPVVAPPS-TGLTDHLTPMGNWM---------LCNDV 64
T_vivax MSMSDSADAKLMLPPSLGFDSSGDLCAPPACRSMQRSSGYSVSGASVSPIGAPGTVLDSSITGVAPPSYRTPAWESGTED 80
T_cruzi MRELSYMFSSVSEPRALGAADDGQVIT-----SHSNSSNHHHHNNFYPLYSASGSTKVSNYGGHFE--------NSGNNI 67
B_ayalai --------------------MHADPCEIKCVHG--- 13
C_fasciculata MSALPQLAGRVPSTEAAPAAHEKISDVPLVIADHFS-------NLYTGTDD-----------S----------------- 45
L_seymouri MSTLTQASERVSCAVPHSATHEKSGDIPLAVTNHIS-------SVYTVLDE-----------G----------------- 45
E_monterogeii MSYTAQLQELAVPAVLSLACKEKTDEVQHAFCGDTC-------NGLTATEE-----------G----------------- 45
L_major MSYSTHLREPAPFAGPSLGSKGKGDDFQHPLGGETC-------NGYTATEE-----------G----------------- 45
T_brucei VDSDSPGCGYDDKFGAGDDEVKG-GELYMWVLDPHSRKLRVPLSLMVPTHATANKGAIPSLCLSFLEGHCRHEWCRQAHV 146
T. congolense AASSGDRCCEKTSSNDGAESAAGSGERYIWVLDPLTRKLRVPVSLLVPTQATATTGTVPSLCMSFLEGRCRHQWCRQAHV 144
T_vivax HSRGGNSHSDPEAMASGGNSTQD-GERWIWVLDPLTRKLRVPLSKLVQTQATATTGTVPSLCISFLEGRCRHHWCRQAHV 159
T_cruzi TSMSNKSDVNVNTCGDSGEKGHE-GEKWIWVLDPLTRKLRVPLSQLVHTQATATTGTVPSLCISFLEGRCRHQWCRQAHV 146
B_ayalai ---AQPDV--EEHPSNGS-DEEASEDKWIWVLDPMTRKLRVPIHHIVPTQATSTSGTVPSLCIAYLEGRCRHPWCRQAHV 87
C_fasciculata SRAATTDVSDEKNSSSSSTDEKKEEDKWIWVLDPMTRKLRVPLNMLVPTHATSTPGTVPSLCIAFLEGRCRHAWCRQAHV 125
L_seymouri NKAATTDMSDEKHSSSSSFDDKKGEDKWIWVLDPMTRKLRVPLNMLVPTHATSTPGTVPSLCIAFLEGRCRHAWCRQAHV 125
E_monterogeii SGAPTAEMSDEKQSSSGSSDSKKEEEKWIWVLDPMTRKLRVPLHLLVPTHATSTPGTVPSLCIAFLEGRCRHAWCRQAHV 125
L_major SAAPTAEVSEEKHSSSASSDYKKEDEKWIWVLDPMTRKLRVPLHLLVPTHATSTPGTVPSLCIAFLEGRCRHAWCRQAHV 125
T_brucei PPHVIPMLRQQALNAPTCCRLHKDPHSTIELTDRFKFIRIVG-NEGSYSSRGTNGEQSDLIPAIRVALTVGLQLIIAQSA 225
T. congolense PMHAIPRLRHEALHAPTCCRLHMDPHDTSVLTDRFKFIRVLG-SEGGFSKSDVNGGACDLIPTDRVAQTVGLLRFIANCS 223
T_vivax QPSAIMQLRHDALHAPTCCRVHDDPHDTSLLTNTYKYIRIVNGAGNECSVLDSCNDQKDLIPTDRVAQTVGLLRFITHYV 239
T_cruzi LPSAIPQLRHEALHAPTCCRLHDDPHDTSVLTSRFKYIRVVN-NNNNNPIGENEHDSSDLISTDRVAQTVGLLRFMAHSV 225
B_ayalai QPSAIPQLRYEALNAPTCCHLHNDPHNISMLTNRFRYIRISN---------STSSGNSELIPADRVASTVGLLRYLVHHV 158
C_fasciculata LPSAIPQLRYEALSAPTCCHFHHDPQDISMLTSHFKFVRITG------------NGGNNELIPAIRIACTVGLRRYLVHNT 194
L_seymouri LPSAIPQLRYEALSAPTCCHFHHDPQDISMLTDHFKFVRIIG------------SGGNNELIPTDRIACTVGLRRYLVHNT 194
E_monterogeii VPSAIPHLRYEALSSPTCCHYHQDPQDISMLTNHFRYVLITG------------NGGSNEQIPADRVACTVGLRRYLVHNV 194
L_major VPSAIPQLRYEALSSPTCCHFHQDPQDISMLKNHFKYVLITG------------NGGSNELIPADRVACTVGLRRYLVHNV 194

```
T_brucei       QAAKEKEEQ-----SQSLGETNQG---------------------AANGAKVERKDVLDVPAKDVCRLH-ISRLCRYVE 277
T. congolense  HSPKEKGRQQEKQSSASPGENGNGSESAEAGRSPYVGR----PANYVTNDAKLDQSMVLDLPAKLVCRLH-LSHRCRYLE 298
T_vivax        SPPKGKGH----QGAANDKNDSQSEGVRETREDVGKSPNIDEDRCCANDSGGESRDDVLDLPAKLICRLH-LAHRCRYLE 314
T_cruzi        PSPKKRVHS---QLSSKEGSNSRSGNIRDTAGEADKSP----GKATSNDRD-DDRELVLDLPAKLICRLHSLSHRCRYLE 297
B_ayalai       PKKKVASDSS----VRGDGAGTSD---------------------------EMDEAQKILDLPAKFICRLH-LAHRCRYLE 207
C_fasciculata  PKKPAEHGD-----PAQPAPGHAS---------------------------EDADKDGILDLPAKFICRLH-LAHRCRYLD 242
L_seymouri     PKKPTDT--------STAASEHKT---------------------------EGFDKDGILDLPARFICRLH-LAHRCRYLD 239
E_monterogeii  PKKSARG--------SNNGAEKRS---------------------------APDESD-VLDLPAKFICRLH-LAHRCRYLD 238
L_major        PKKSACA--------SINAAEKRS---------------------------VPDEND-ILDLPAKFICRLH-LAHRCRYLD 238

T_brucei       DCKNIHICREYDLQLPPPPNLVCLLNSITPSMTMINIGERSYSSTMLSLGEVIDEVFNIICDQQRRSVMNNTPAAR-MTP 356
T. congolense  DCNNIHICREYELHLQPPPHLTAALSSVMPTTTTITIGDRNYSSTMISLGDVIDEVFRIICDQQRQCALTSVPAAGSAMP 378
T_vivax        DCNNIHICREYELRLQPPPQMVAALSAITPATRIATVCERRYTVTMLSDGEVIDGTFRMICDQQRQSVLPLSSQSQ---- 390
T_cruzi        DCNNIHICRECELRLQPPSHVMAILSNVAPGTRTITVGDTCYAATQLAVGEVIDEEFRAICDQQRHAVATRSWDES---- 373
B_ayalai       DCNNIHICREYELRLQQPPHMLNALASITTTTRTVIIADICYAITPLAVGEVSDEDFDAIRGAQR----EATNCRFATCW 283
C_fasciculata  DCNNIHICREFEVRLQPPPQMLSSLNSVTTSTRTVNIGDTCYTVTPLAVGDVSDDDFNAIAEAQK-INHRNSGTPASTPF 321
L_seymouri     DCNNIHICREFEVRLQPPPQALSSLNSVTTSTRTVNIGDTCYTVTPLAVGDVSDDDFNAIVEAQK-INHRNAGTPASTAF 318
E_monterogeii  DCNNIHICREFEVRLQPPPQILSSLNSVTTSTRTVSIGDTCYTVTPLAVGDVSDEDFNFIVETKKKASHCNAAMLPPAAF 318
L_major        DCNNIHICREFEVRLQPPPQILSSLNSVTTSTRTVSISDTCYTVTPLAVGDVSDDDFNAIAEAKR-INHRNASTPASATF 317

T_brucei       ASQPYQHANQVVSCANPGVDAAINSASPLSLGDAN----LYTAPRGGTSPASCGMGDATPFAPASQRSLGQNGSPNLGAA 432
T. congolense  PPSLHSHASPMIFDGSRPVDVAERITSPLSLEDTSP-RTLFCNSMGSAMPAAHG-G--AVYVPATDLSPPQGSSPVLAAT 454
T_vivax        -QSCSGPRQ------------KSESVSSLEAGAPP--FVLESAVQPND--NSNANNRNCVFTTRSEGARSHANSPTLG-A 452
T_cruzi        -PSMAPHRSPLIYSGSGASSFTGASVSPPTFSTGK--WGLESKVNAAATTNSAAHCGPMLFPGNPETGQSDGDSPIAAGA 450
B_ayalai       QGPVPAYDSTATSS---------CGNSPMMYSAGTG-RVSPTTSATGANSGTFPAIPDSFGLNPCSSIPGHSSLTDSMLT 353
C_fasciculata  WSVAPPLDSAFGGS---------ERSSPLLYSGAGGVGAFPAAPDYRLAQRTPN--SSVTTLNGHTPTFVHTESPSHQPQ 390
L_seymouri     LNTALQLDSAIVGS---------EGSSPLIYSGSGGASVFPASPDYRMTQRTPN--GSLTAHAGQSPAFTYVESPMQQLQ 387
E_monterogeii  KNLAPPFDATLGDSS--------GRGSPMSYTTAG--SGFPALPEYWCALRTPSSSTAVTPLNGPSLGFLHTESSSQIQQ 388
L_major        KSAIPPYDAALGGSS--------ENGSPMPYSGAG--GAFPPFPDYRGALRTPN-TSAVTPPSGKSPAFLHVESPSQIPP 386

T_brucei       AA----------GSSNVTRVLRIFDVRPKSAGEGGRERGSTHRGHNTNGNGSAGASPFLNGRGSES---ASRSRNGASVS 499
T. congolense  AS----------SPQCISRVLRIYDVRPKSAAPSGQGKSGSYRSPGTLINGSAGNSPLMNGWKGTS---VNNSYNSVGSS 521
T_vivax        IPHGAPIPVSHNTPQQVARVLRIYDVRPRGGQGNASTVRGADNTSSCSNNNNNGSSNGTNTACSTPYLGASRVGVTGGST 532
T_cruzi        PPQGGP----HHTSEAVTRALRIYDVRPKAAQNNAS-----NNHFASSNIGGSGNSIVKN-SMG------------GSGA 508
B_ayalai       KA------------AGVR-TLRIYDVRGKIPGSHAQTSRK-SQDLHRQSTTTSTSDAATAPMCHPS----------RQCS 409
C_fasciculata  ----------------SFSGHLRIYDVRPKPQTDTAASSCTNSPALNPNTTEKAAAG-KLPPPKYAD----------CSSP 444
L_seymouri     ----------------NFSDHLRIYDVRPKPQTETAASSCTNSPALNPNSAEKAGAG-KLPPPKYAD----------CLSP 441
E_monterogeii  GG------------LNFGGHLRVYDVRTKAQSDTATPSCTNSPAVNPSVLEKSGVGSKLTLPKYAG----------NASP 446
L_major        GG------------FGFGGHLRVYDVRPKNQSDTVTPSCTNSPAMNPNTQEKNGAGGKIAPPKYTD----------GASP 444

T_brucei       YNNYTCPNSKNGSGNGCAAGTG------------VPPCGINGSDRNACSGS----HRRGSRHLTGQSNNSG--------- 554
T. congolense  HSHHGNPSNESNDGNGHCGVTGGSRPDGMQLGEQRSSANSNNNKRSDCGNNGPNHHRRHHKQQKQQHAQSQQQS---PLI 598
T_vivax        HGSKSGRDGKRNSGSSVGAGSP------------KHGSRGVGQQHHDHSQRYHGQQQQHHYHNHHHHHHQSSHSKSGKHT 600
T_cruzi        KNNCSGGGGGHNSG----AGTK------------HNGSGGNG--------GFRGANGSSGYHHSHGHHAN------RHT 558
B_ayalai       VPVASLKCPRETTNTSS--TDA-----------------GVS-------------HSTSGVVTPMAEG----------- 445
C_fasciculata  STSVNRSAGLSVGGCLSNSTDG-----------------GVS-------------LGTSGANTPVADGGYV--------- 485
L_seymouri     SASMNRSVGLSAGGCLSNSTDG-----------------GVS-------------GGTSGANTPLEDGGYV--------- 482
E_monterogeii  TALVTRSNGMSASGCAS------------------------------------LVASEVNTPLTTGRHS--------- 479
L_major        PAMASRSNGMSGSGCASSRTEG-----------------GMS-------------LATSEANTPLATGGYS--------- 485

T_brucei       --RGSAKRRQKA      564
T. congolense  QLRGNACSHRSP      610
T_vivax        AFTGSPVMTSTQQ     613
T_cruzi        ALKG---INS        565
B_ayalai       --ASSSA           450
C_fasciculata  PFSGRAALAQKK      497
L_seymouri     PFSSRTAVSLKK      494
E_monterogeii  IMSRPVAVTGRK      491
L_major        IISGRTAVTTRK      497
```

CCCH motif

Grey: Matches the concensus group. a=(DE) b=(HKR), f=(AGILV), m=(NQ), o=(FWY), h=(ST), i=(P), s=(CM).

Black: residues that match the Consensus exactly
