# Supplementary figures and images for: Stress susceptibility in Trypanosoma brucei lacking the RNA-binding protein ZC3H30
Source: PLoS Negl Trop Dis. 2018 Oct 1;12(10):e0006835. doi: 10.1371/journal.pntd.0006835 (PMC6181440; doi:10.1371/journal.pntd.0006835)

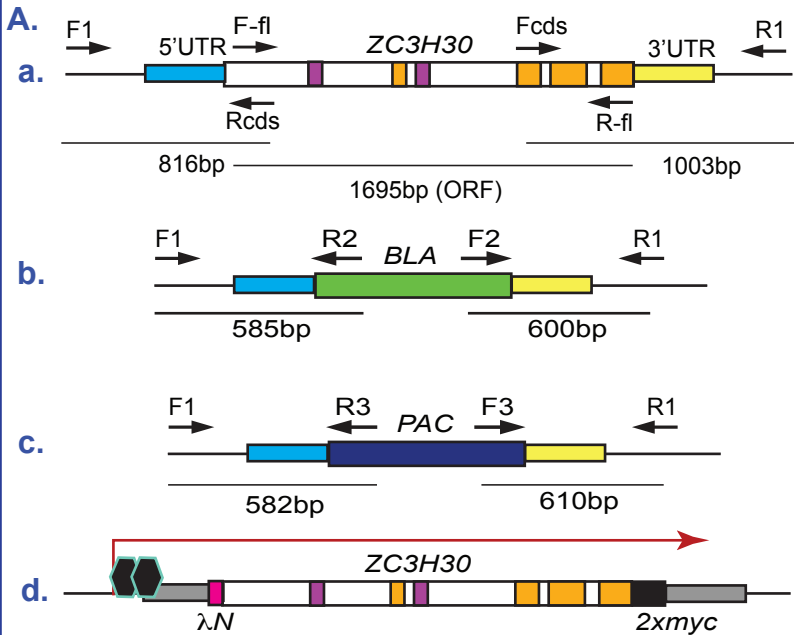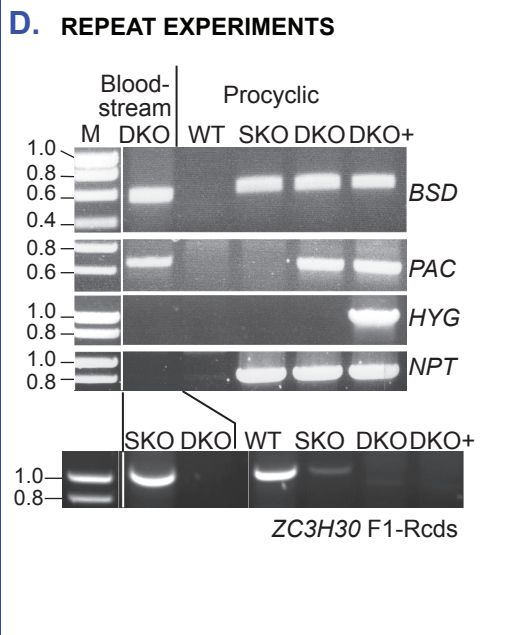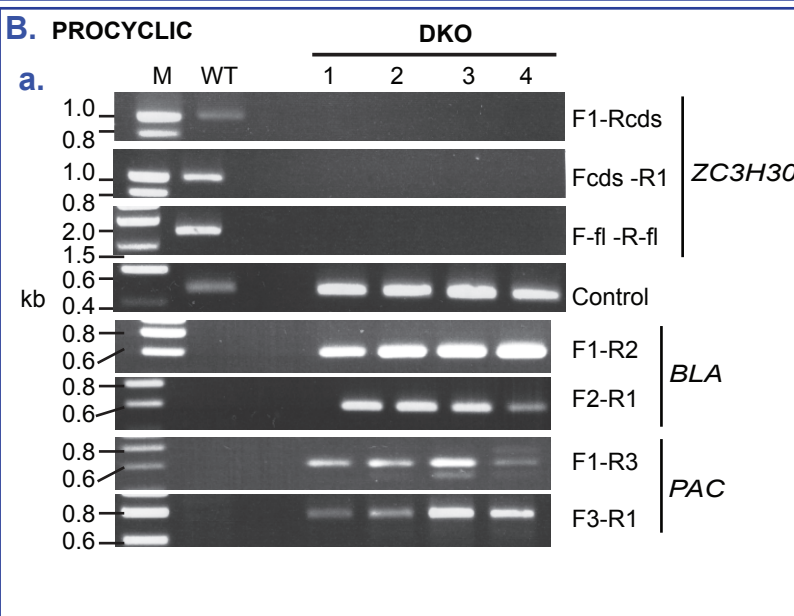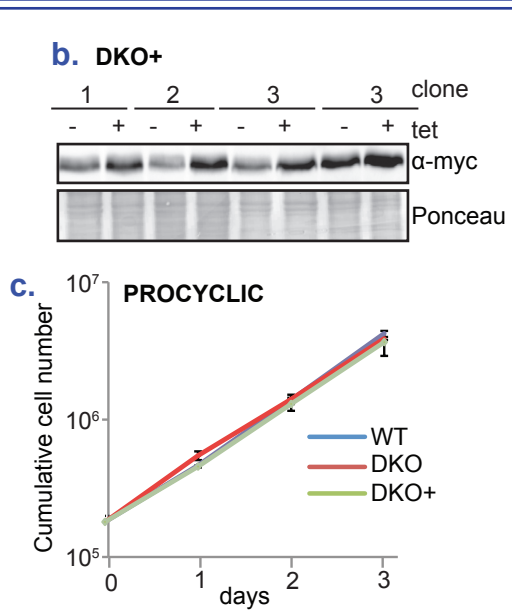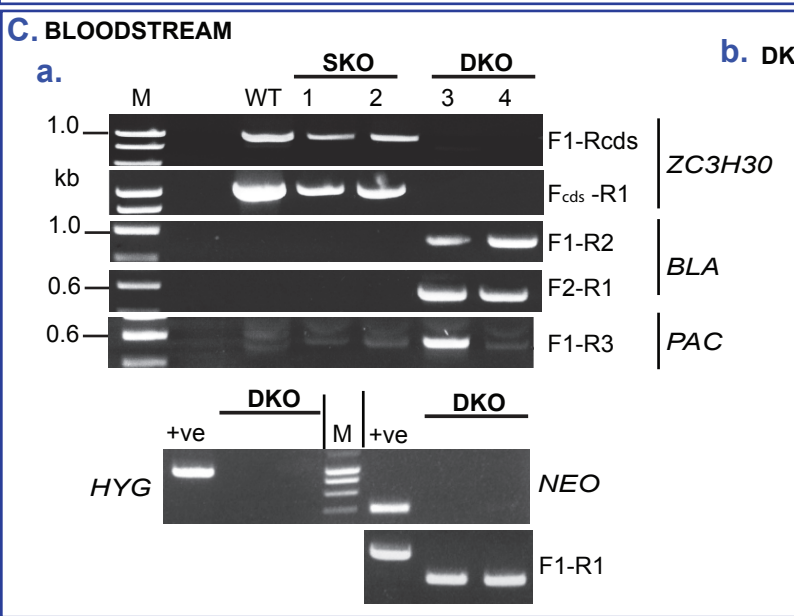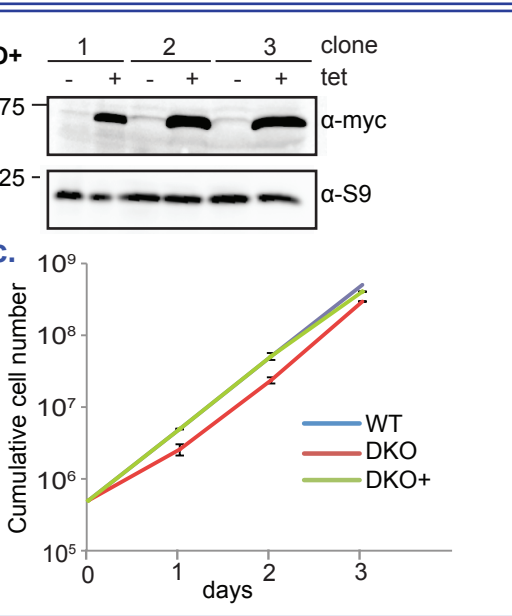

Supplement: S2 Fig — A) Schematic representation of ZC3H30 alleles (a), the resistance cassettes used to replace them by homologous recombination (b,c) and the indiucible lambdaN-ZC3H30-myc cassette (d). Only the ZC3H30 coding region is to scale. Primer locations are indicated by small arrows and sizes of PCR products are also shown. B) Knockout in procyclic forms. (a) Ethidium-bromide-stained agarose gel pictures for PCR products shown in (A); (b) Western blot showing expression of lambdaN-ZC3H30-myc in DKO+ lines with and without tetracycline; (c) growth of three lines, each measured in duplicate, with cumulative counts shown as mean ± standard deviation. C) As (B), but for the knock-out in bloodstream forms. "SKO" is the single knock-out line with only puromycin resistance. For unknown reasons, then PAC amplification failed in all but one preparation. D) Repeat PCRs done for the procyclic cell lines and an independent knockout in bloodstream forms. (PDF) [file pntd.0006835.s002.pdf]

**A.**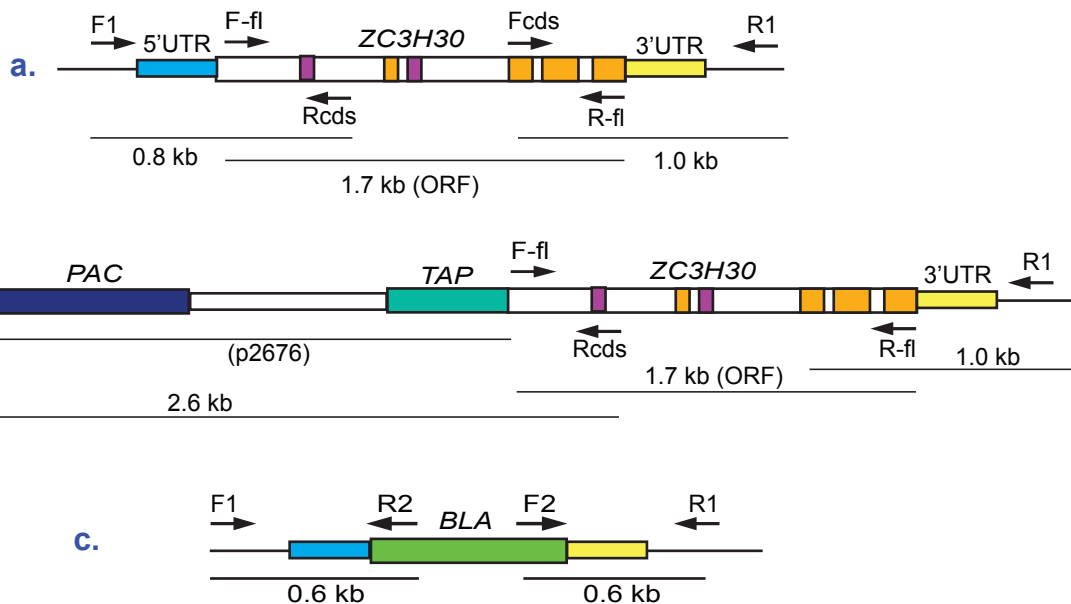**B.**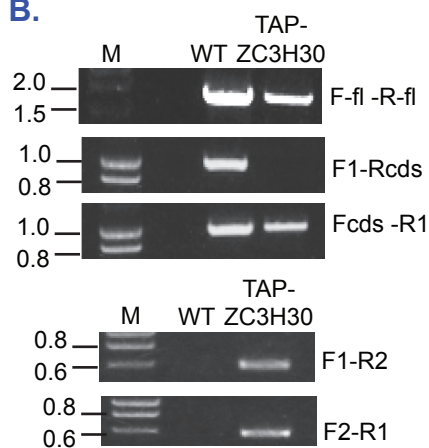**C.**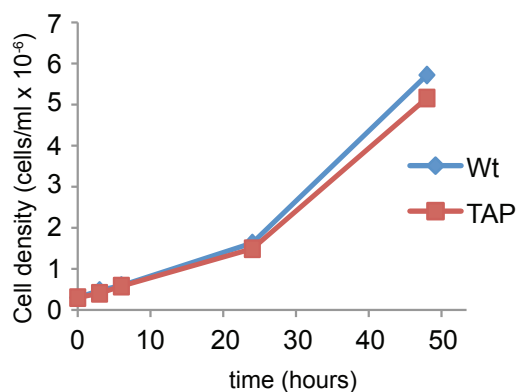**D.**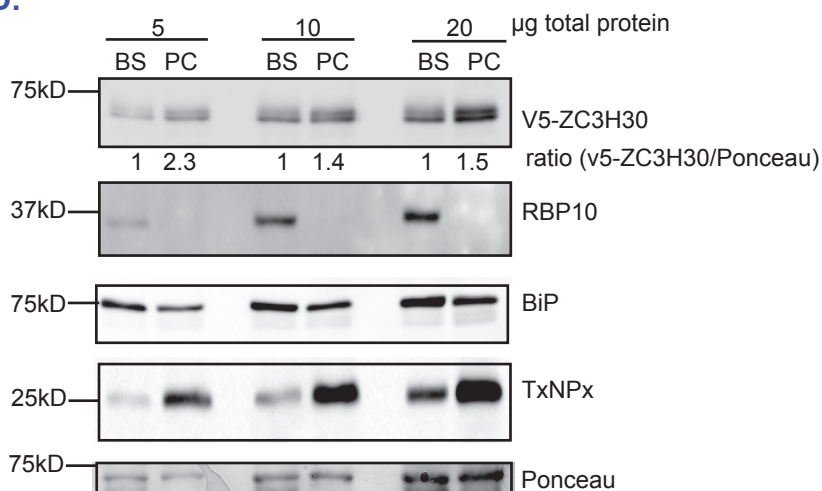

Supplement: S3 Fig — A) (a) Schematic representation of ZC3H30 alleles; (b) the in situ TAP-tagged allele; and (c) the integrated blasticidin resistance cassette. Only the ZC3H30 coding region is to scale. Primer locations are indicated by small arrows and sizes of PCR products are indicated. B) Ethidium-bromide-stained agarose gel pictures for PCR products shown in (A). In the cell line expressing only TAP-ZC3H30, the PCR product for F1-Rcds should be 2.6 kb but no band was obtained. Presumably the PCR conditions were inappropriate for this particular product. Successful expression was however seen both by Western blotting (Fig 6A) and mass spectrometry (S4 Table). C) Growth of cells expressing only TAP-ZC3H30 after a heat shock (1h, 41°C) compared with WT. D) Expression of V5-ZC3H30 in cell lines with one in situ-tagged copy and one Wt copy of the gene. Western blots were incubated with antobodies detecting the indicated proteins. (PDF) [file pntd.0006835.s003.pdf]

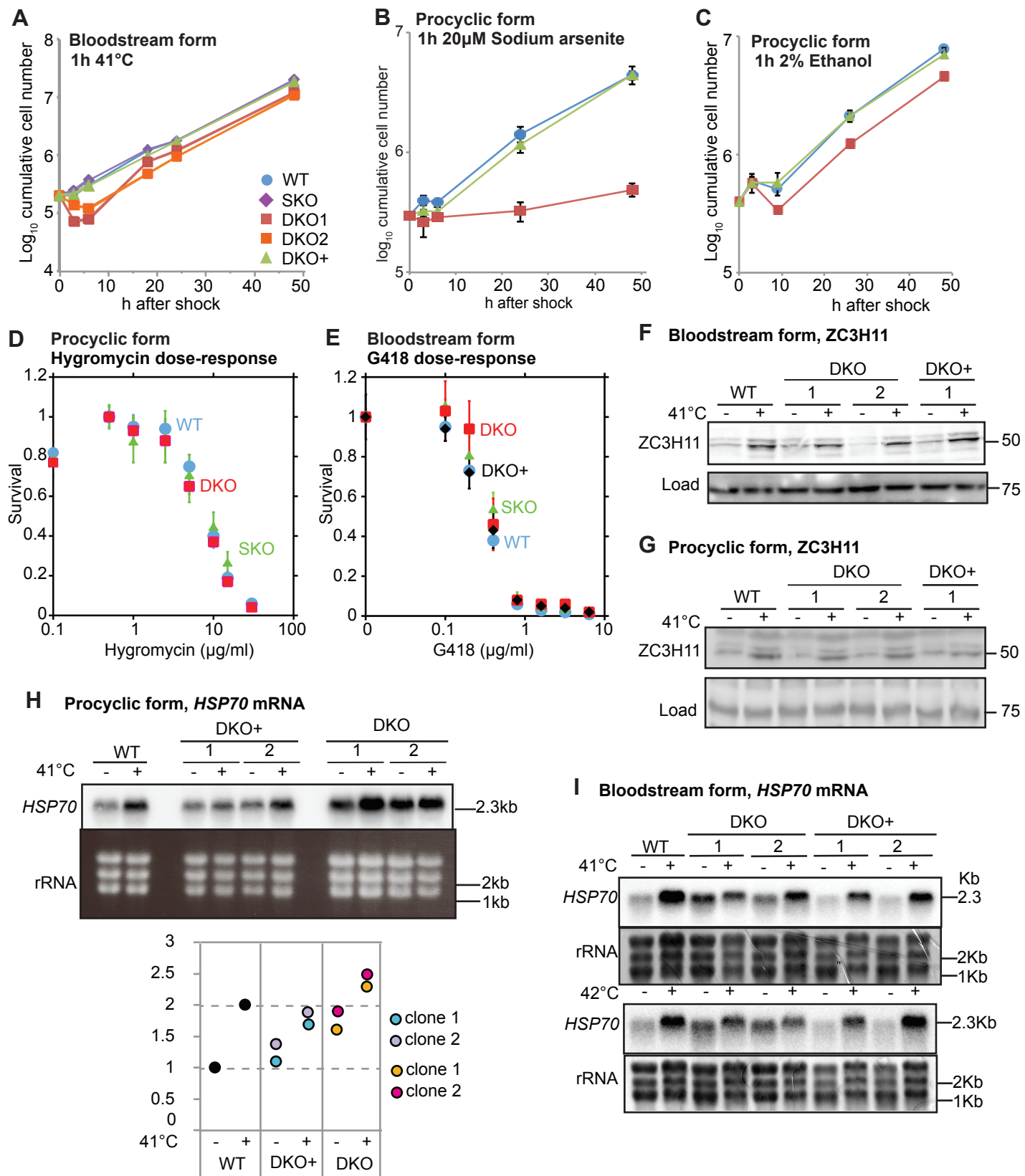

Supplement: S4 Fig — A. Growth of bloodstream forms after 1h at 41°C. This was a single experiment, individual data points are shown. B. Growth of procyclic forms after 1h in 20μM sodium arsenite. This is combined data from two experiments. Experiment 1 had time points 3h, 6h, 24h and 48h, with 1x WT, 1x DKO, and 2x DKO+. Experiment 2 had time points 3h, 24h and 48h, with 2x WT, 4x DKO, and 2x DKO+. C. Growth of procyclic forms after 1h in 2% ethanol. This is a combination of two independent experiments. Experiment 1 had time points 3h, 24h and 48h, with 2x WT, 6x DKO, and 4x DKO+. Experiment 2 had time points 9h, and 28h, with 1x WT, 3x DKO, and 3x DKO+. For the graph, the data from the 24h and 28h have been put together and placed at 26h. D, E) Dose-response curves for procyclic forms grown with hygromycin (D), and bloodstream forms grown with G418 (E). F, G) Expression of ZC3H11 in bloodstream (F) and procyclic (G) forms after heat shock, detected by Western blotting. Using total cell lysates, ZC3H11 is normally obscured by background from tubulin. Cells are therefore fractionated to remove triton-insoluble cytoskeletons, before SDS-PAGE [27]. The background bands on the Western blots originate from residual cytoskeletal proteins and can be used as the loading control ("Load"). H) Levels of HSP70 mRNA in procyclic forms with and without heat shock. Quantitation of the blot signals relative to WT is shown below. The experiment was not repeated because we instead subjected RNA to RNASeq and could not detect any effect of ZC3H30 on HSP70 mRNA. I) Levels of HSP70 mRNA in bloodstream forms with and without heat shock. These blots are quantitated in Fig 5. (PDF) [file pntd.0006835.s004.pdf]

**A. Polysome profile: 27°C**

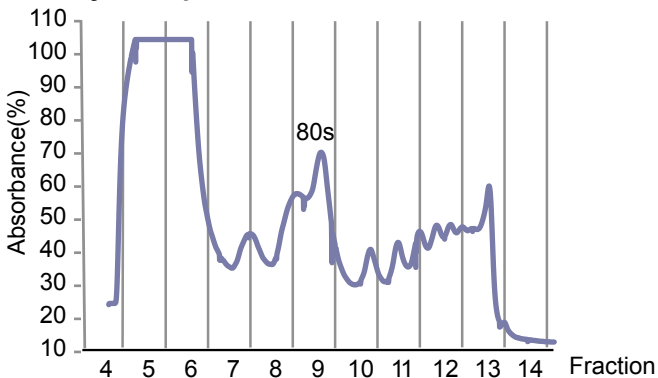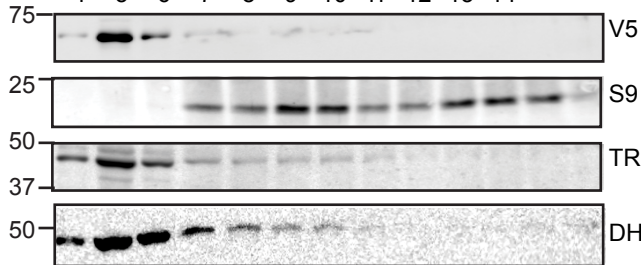

**B. Polysome profile: 1h 37°C**

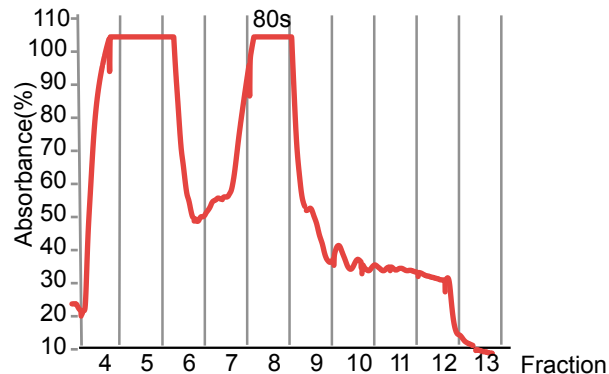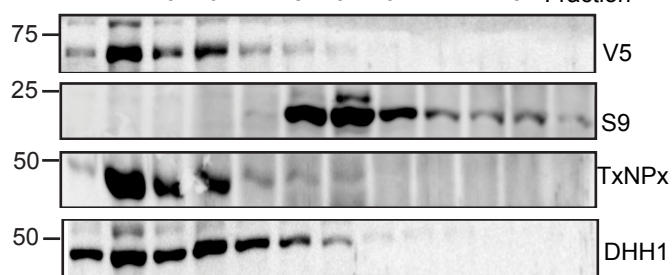

Supplement: S5 Fig — A) Extracts from cells expressing in situ V5-tagged ZC3H30 were fractionated on sucrose gradients; the upper panel shows absorbance at 254 nm (arbitrary units) and the lower panels are Western blots probed with antibodies as indicated. S9 is ribosomal protein S9 and TR is trypanothione reductase; relevant marker molecular weights (in kDa) are also indicated. The least dense fractions are on the left. B) As (A) but after a 39°C heat shock; TxNPx is tryparedoxin peroxidase. (PDF) [file pntd.0006835.s005.pdf]

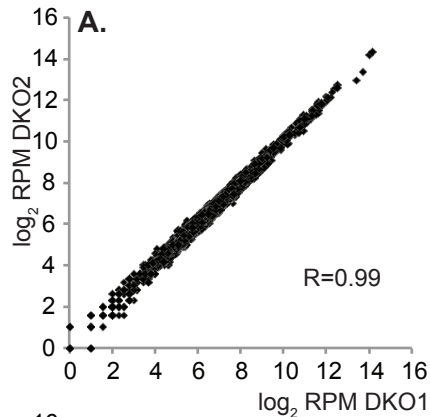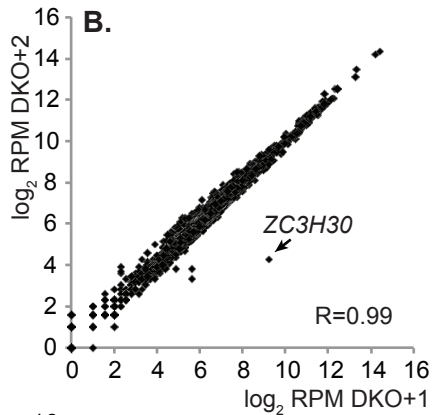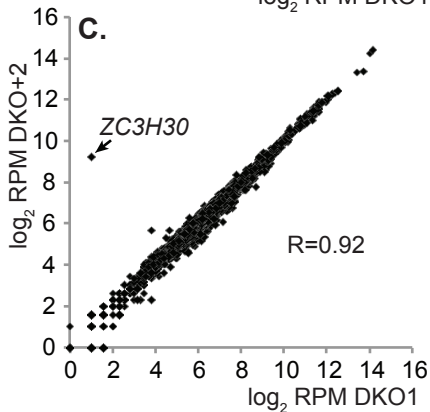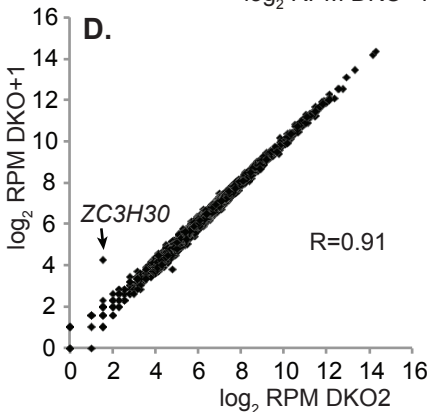

Supplement: S6 Fig — Each spot represents a gene. Data are in S2 Table. Panels A, B, C and D are comparisons of different paired datasets as indicated on the "x" and "y" axes. (PDF) [file pntd.0006835.s006.pdf]

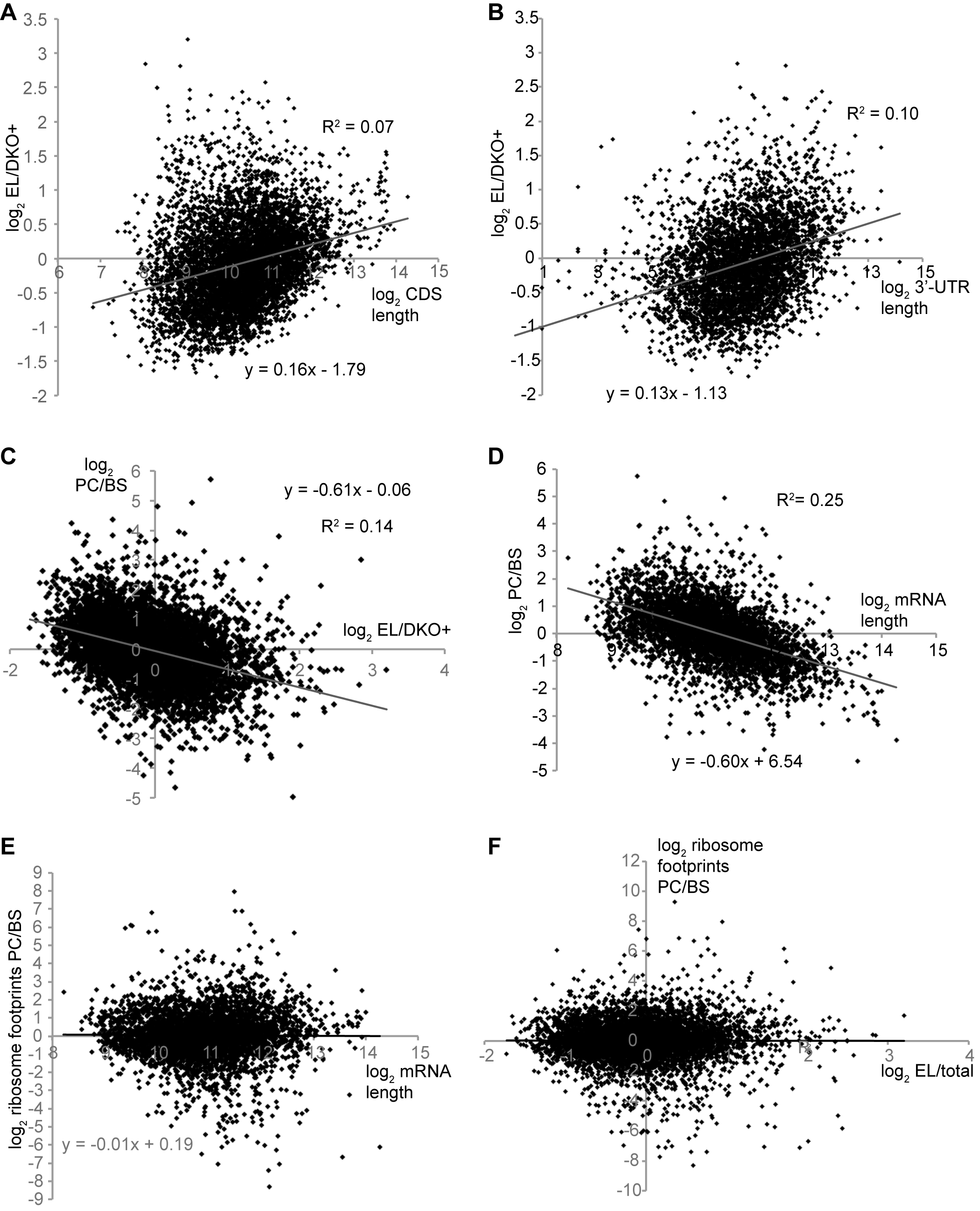

Supplement: S7 Fig — In each plot, each spot represents a single unique gene. A. There was no correlation between TAP-ZC3H30 binding and mRNA coding region (CDS) length. On the y-axis, we have plotted (on a log2 scale) the average RPM from both eluates divided by the average RPM from DKO+ cells. In all graphs, regression lines and correlation coefficients were calculated in Microsoft Excel. B) As (A), except that this is the relationship between binding and mRNA 3'-UTR length. C) Relationship between binding and developmental regulation of mRNA abundance [44]. The y-axis shows the log2-transformed ratio between procylic- and bloodstream-form expression. D) Relationship between mRNA length and developmental regulation of mRNA abundance. E) Relationship between mRNA length and developmental regulation of total ribosome footprints [42]. F) Relationship between binding to ZC3H30 and developmental regulation of total ribosome footprints [42]. (TIF) [file pntd.0006835.s007.tif]

**A**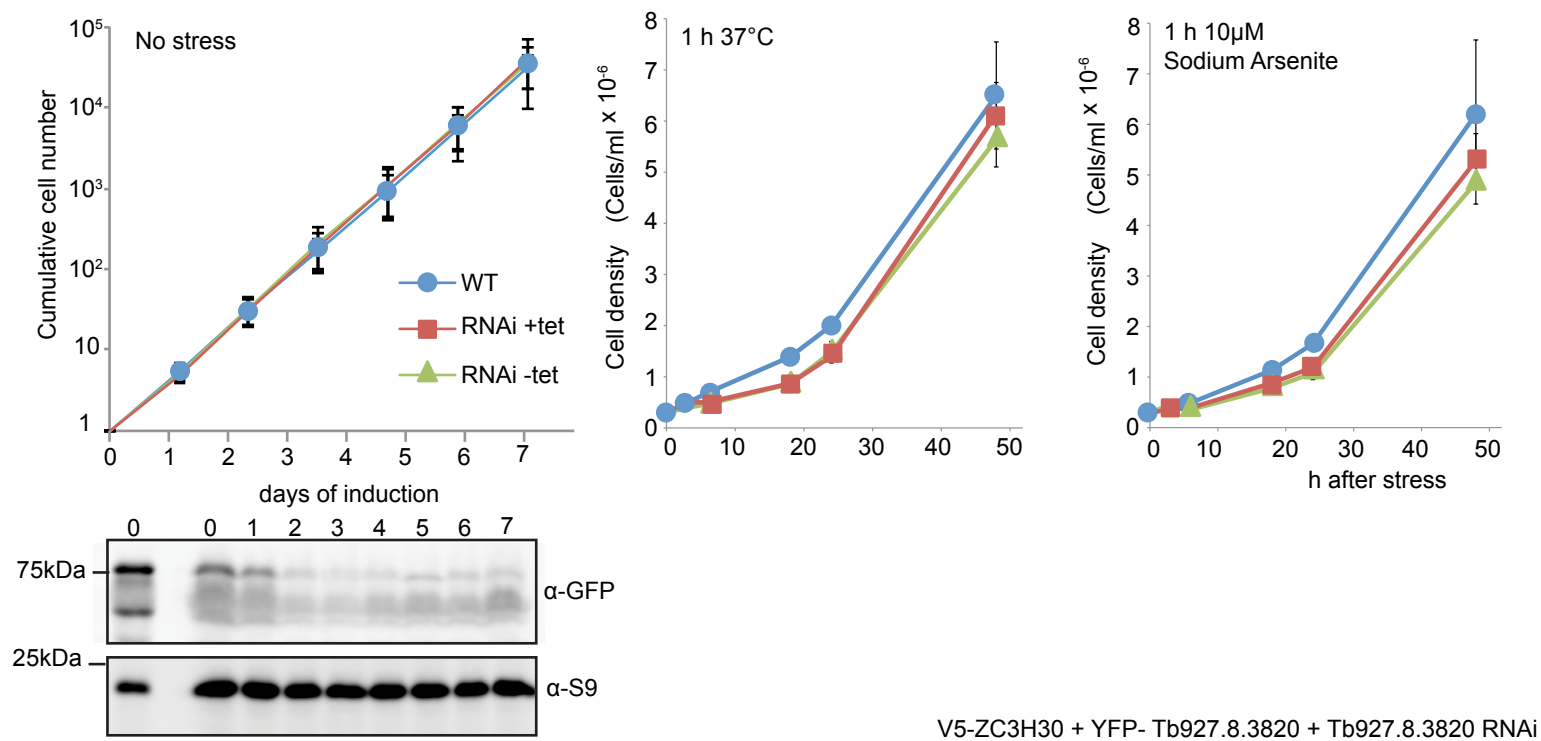**B**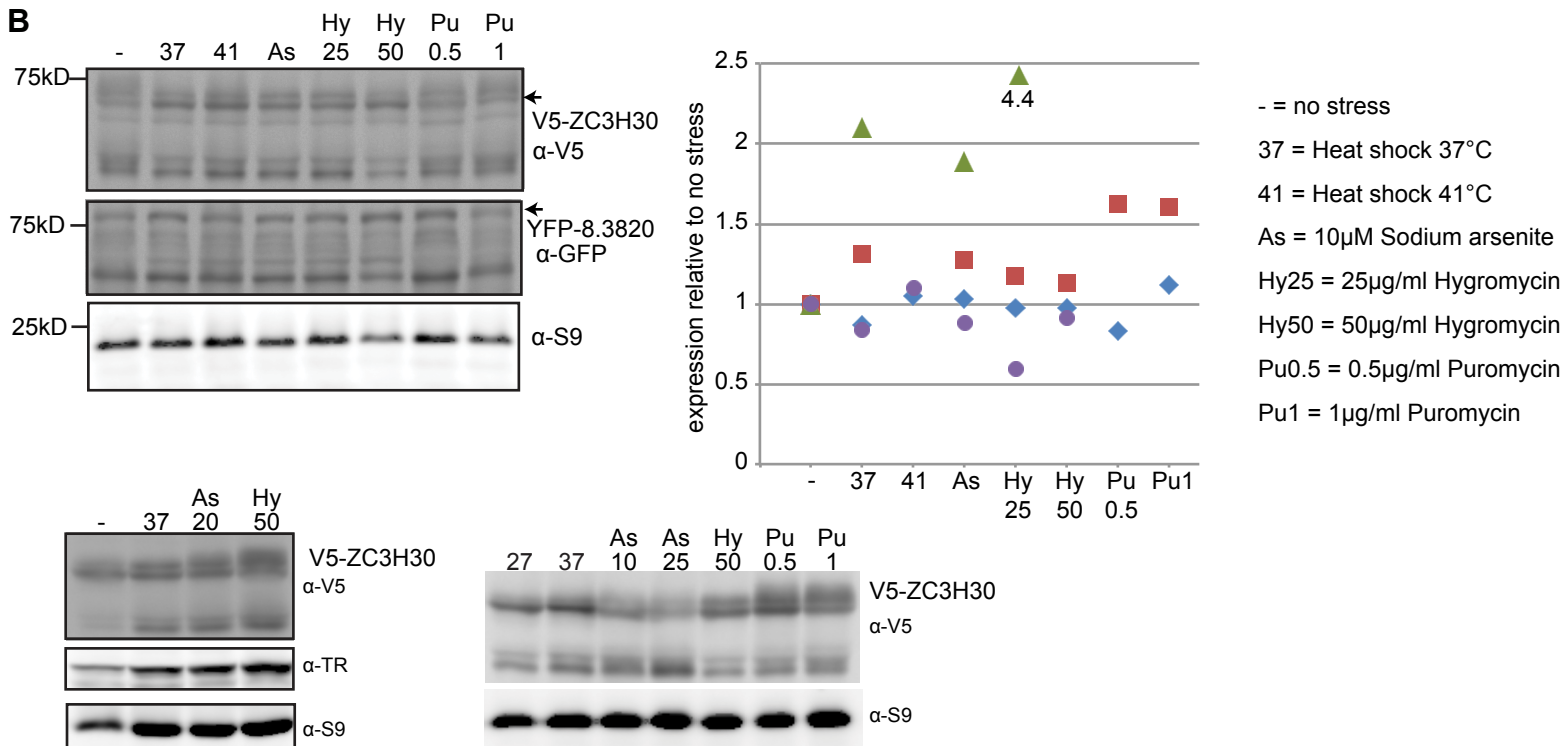

Supplement: S8 Fig — A) Growth of cells with RNAi under normal conditions, or after arsenite or a mild heat shock. The RNAi was done in cells expressing YFP-927.8.3820 and the equivalent Western blot is shown beneath the left-hand plot. The top band has the expected size but we usually saw a second band, which might be a degradation product. B) Effects of various stresses on the abundances of V5-ZC3H30 and YFP-8.3820 in procyclic forms. Sample blots are shown and quantifications for 2–4 measurements of V5-ZC3H30 are shown on the right. There was also no significant change in YFP-8.382 after stress. A second band of V5-ZC3H30 was routinely seen. Arrows indicate the migration of the full-length proteins. (PDF) [file pntd.0006835.s008.pdf]
